# The Molecular Phenotype of Endocapillary Proliferation: Novel Therapeutic Targets for IgA Nephropathy

**DOI:** 10.1371/journal.pone.0103413

**Published:** 2014-08-18

**Authors:** Jeffrey B. Hodgin, Celine C. Berthier, Rohan John, Elisabeth Grone, Stefan Porubsky, Hermann-Josef Gröne, Andrew M. Herzenberg, James W. Scholey, Michelle Hladunewich, Daniel C. Cattran, Matthias Kretzler, Heather N. Reich

**Affiliations:** 1 Department of Pathology, University of Michigan, Ann Arbor, Michigan, United States of America; 2 Department of Internal Medicine, Nephrology, and Computational Medicine and Bioinformatics, University of Michigan, Ann Arbor, Michigan, United States of America; 3 Department of Laboratory Medicine and Pathology, University Health Network and University of Toronto, Ontario, Canada; 4 Department of Cellular and Molecular Pathology, German Cancer Research Center, Heidelberg, Germany; 5 Institute of Pathology, University Medical Center Mannheim, University of Heidelberg, Mannheim, Germany; 6 Department of Nephrology, Sunnybrook Health Sciences Center, Toronto, Ontario, Canada; 7 Department of Laboratory Medicine and Pathology, University Health Network and University of Toronto, Toronto, Ontario, Canada; 8 Department of Medicine, University Health Network and University of Toronto, and the Toronto Glomerulonephritis Registry, Toronto, Ontario, Canada; University of Florida, United States of America

## Abstract

IgA nephropathy (IgAN) is a clinically and pathologically heterogeneous disease. Endocapillary proliferation is associated with higher risk of progressive disease, and clinical studies suggest that corticosteroids mitigate this risk. However, corticosteroids are associated with protean cellular effects and significant toxicity. Furthermore the precise mechanism by which they modulate kidney injury in IgAN is not well delineated. To better understand molecular pathways involved in the development of endocapillary proliferation and to identify novel specific therapeutic targets, we evaluated the glomerular transcriptome of microdissected kidney biopsies from 22 patients with IgAN. Endocapillary proliferation was defined according to the Oxford scoring system independently by 3 nephropathologists. We analyzed mRNA expression using microarrays and identified transcripts differentially expressed in patients with endocapillary proliferation compared to IgAN without endocapillary lesions. Next, we employed both transcription factor analysis and in silico drug screening and confirmed that the endocapillary proliferation transcriptome is significantly enriched with pathways that can be impacted by corticosteroids. With this approach we also identified novel therapeutic targets and bioactive small molecules that may be considered for therapeutic trials for the treatment of IgAN, including resveratrol and hydroquinine. In summary, we have defined the distinct molecular profile of a pathologic phenotype associated with progressive renal insufficiency in IgAN. Exploration of the pathways associated with endocapillary proliferation confirms a molecular basis for the clinical effectiveness of corticosteroids in this subgroup of IgAN, and elucidates new therapeutic strategies for IgAN.

## Introduction

IgA nephropathy (IgAN) is the most common cause of primary kidney disease globally and up to one third of patients with IgAN will progress to kidney failure by 10 years following diagnosis [1], [2]. IgA nephropathy is a clinically and pathologically heterogeneous disease. A gap in understanding of the molecular mechanisms underlying the development of progressive disease has precluded identification of any specific targeted therapy.

A new pathologic classification system for IgA nephropathy has recently been proposed. Through a rigorous international collaborative effort, reproducible morphologic features in IgAN independently correlated with long-term renal disease outcome were identified [3], represented by the “Oxford MEST score”. We have previously shown in two independent cohorts, that the pathologic finding of glomerular endocapillary proliferation (E1 in the Oxford schema) in kidney biopsies of patients with IgAN is associated with a higher degree of proteinuria and impaired renal function at the time of biopsy [4], [5]. This lesion is also associated with a more rapid rate of loss of kidney function particularly in patients who do not receive immunotherapy [6], [7]. Importantly, clinical data suggest that this lesion may be amenable to immunomodulatory therapy. The relationship between endocapillary proliferation and kidney failure is mitigated by treatment with corticosteroids [4], [8], [9], [10], [11]. Improvement of endocapillary proliferation is documented in serial biopsies of corticosteroid-treated patients [12].

Corticosteroids modulate activity of several transcription factors, and their main effects on immune responses are ascribed to inhibition of the activity of nuclear factor κ-B (NFκB) [13], [14]. Unfortunately, due to the protean cellular effects of corticosteroids, this therapy is associated with a broad range of toxicity, therefore more targeted treatment options that influence development of endocapillary proliferation are urgently needed.

To determine the molecular pathways involved in the development of endocapillary proliferation in patients with IgAN, and to identify novel therapeutic targets, we evaluated the glomerular transcriptome of microdissected kidney biopsies from patients with IgAN. We compared the mRNA expression profile of biopsies with glomerular endocapillary proliferation (E1) to biopsies without endocapillary proliferation (E0), based upon interpretation by three independent nephropathologists. We then identified the mRNA transcriptomic profile associated with the pathologic finding of endocapillary proliferation in IgAN. Next, we employed both transcription factor analysis and in silico drug screening and confirmed that the endocapillary proliferation transcriptome is enriched with pathways modulated by corticosteroid exposure. With this approach we also identified new therapeutic targets that may be responsible for the pathogenesis of this lesion, and a panel of small molecules that may be candidates for modulation of the pathways responsible for development of endocapillary proliferation. Taken together, our findings are proof of principle that evaluation of the human tissue molecular phenotype associated with a distinct, clinically important and carefully annotated pathologic phenotype can yield insights regarding novel therapeutic strategies for the treatment of IgAN.

## Materials and Methods

### Kidney biopsy samples

Biopsy material was obtained from the European Renal cDNA bank (ERCB) [15] (n = 16) and the Toronto Glomerulonephritis Registry biobank (n = 6). After written informed consent for the clinical procedure, a section of the clinical biopsy core is stored in RNAlater (Life Technologies, Carlsbad CA) at 4C. Once determined that this tissue is not required for clinical diagnosis, this core segment is available for research purposes as per the institutional (University Health Network) and ERCB informed consent process. Tissue and data access are approved locally at ERCB [15] site contributors and the University Health Network (UHN REB 07-0717-T, 11-0748-BE). All samples were obtained from patients who had provided written informed consent to these protocols via the described ERCB or University Health Network procedures. Renal function of patients was estimated using the CKD-EPI equation [16].

### RNA extraction and microarray hybridization

For both cohorts, the RNA was extracted from the microdissected glomerular compartment of kidney biopsies using identical protocols as previously described [15], [17]. Amplification of RNA was performed with the GeneChip (Affymetrix, Santa Clara, CA) two-cycle amplification protocol and the NuGEN protocol (NuGEN San Carolos, CA). Corresponding cDNA was hybridized to Affymetrix Human U133A Genechips (Affymetrix, Santa Clara, CA) for the ERCB cohort and Affymetrix Human U133 Plus 2.0 Genechips for the Toronto cohort and processed according to the manufacturer's instructions.

### Scoring of glomerular histological lesions

A single PAS-stained section from each case was reviewed by four nephropathologists (JH, RJ HJG, and SP) blinded to the reported diagnosis, and the glomeruli were scored according to the Oxford MEST scoring system [3], [4] (Table S1). In the case of discrepancy in the designation of endocapillary proliferation score, two pathologists reviewed the pathology a second time and determined a consensus diagnostic score.

### Microarray data processing, analysis and pathway mapping

Microarray data normalization and filtering were performed as previously described [18] using the version 15 of the Human Entrez Gene custom CDF annotation (http://brainarray.mbni.med.umich.edu/Brainarray/default.asp). Microarray gene expression data of the ERCB and Toronto cohorts were combined and batch-corrected using the Combat method implemented in the GenePattern pipeline (http://www.GenePattern.org). Principal component analysis using ArrayTrack software (http://www.fda.gov/ArrayTrack) supported the use of the merged ERCB and Toronto mRNA expression profiles for further analysis.

Differential mRNA expression between the E1 (n = 7, 5 from the ERCB and 2 from the Toronto cohort) and E0 (n = 15, 11 from the ERCB cohort and n = 4 for the Toronto cohort) biopsies was evaluated using the Significance Analysis of Microarrays (SAM) method implemented in the TIGR MultiExperiment Viewer (TMEV) application [19]. Genes regulated between two groups with a q-value (depicting the false discovery rate) <0.05 were considered significant. Transcript expression was correlated with clinical variables using Pearson correlation, and adjustment for multiple testing was performed using a false-discovery-rate adjusted p value.

The functional context of significantly regulated genes was studied by creating biological literature-based networks using Genomatix Pathway System software (GePS) (www.genomatix.de). Canonical pathways were analyzed using Ingenuity Pathway Analysis software (IPA) (www.ingenuity.com). Gene expression datasets are available on Gene Expression Omnibus (GEO) under the accession number GSE50469 (http://www.ncbi.nlm.nih.gov/geo/).

### Connectivity Map analysis and Drug Pair Seeker analysis

Differentially expressed genes with a fold change ≥1.5 for the up-regulated genes and ≤0.6 for the downregulated genes were converted into probeset signatures, imported, and analyzed by Connectivity Map (CMAP) (www.broadinstitute.org/cmap) [20], as well as by the Drug Pair Seeker program (http://www.maayanlab.net/DPS). This database provides access to data regarding in vitro cellular responses of human cell lines to approximately 1300 bioactive compounds. This allows users to identify which compounds would be expected to revert disease-associated gene expression signatures derived from user-provided datasets. For this CMAP analysis the imported query of differentially expressed transcripts was compared with predefined gene expression signatures of therapeutic compounds and ranked according to a connectivity score (+1 to −1) representing relative similarity in regulation to the imported gene list. A filtering step for the “most representative” gene expression signature was employed to manage the large amount of data in the CMAP database. Compounds with negative connectivity scores, representing genes discordantly expressed to the imported query, are predicted to exert biologic effects that revert disease signatures in the renal tissue. Drug Pair Seeker was used to predict and prioritized which drug pairs from CMAP data could be associated together to reverse the direction of gene expression.

### Computational promoter analysis

A list of 1495 known human transcription factors was identified using a published data set [21]. Genomatix software (Genomatix® ElDorado database version 07/2009) was used to identify the transcription factors able to bind in the promoter of the 424 differentially expressed genes (E1 vs. E0).

## Results

### Clinical cohort

After screening for RNA and array expression quality, a total of 22 biopsy samples had sufficient glomeruli for both histological and molecular analysis. Pathologic review revealed 7 biopsies with endocapillary proliferation (E1) and 15 biopsies without endocapillary proliferation (E0). The clinical characteristics of the patients at the time of kidney biopsy are described in Table 1. As observed in previous studies, patients with endocapillary proliferation had a higher degree of proteinuria (2 fold-more) at the time of biopsy (p-value = 0.0090).

**Table 1:** Clinical characteristics of patients at the time of kidney biopsy.

	E1 and E0	E1	E0
n	22	7	15
Gender	15 M/7 F	4 M/3 F	11 M/4 F
Age (years)	42.6 (16.5)	42.0 (22.6)	42.9 (13.6)
Mean arterial pressure (mmHg)	98 (10.6)	99.7 (9.6)	97.3 (11.3)
Serum creatinine (mg/dL)	1.8 (1.7)	2.1 (1.6)	1.6 (1.8)
CKD-EpiGFR (ml/min/1.73 m^2^)**	68.7 (37.5)	61.8 (46.0)	72.2 (33.9)
Median proteinuria (g/day) (min,max)*	2.8 (0.5,10.0)	4.2 (2.0, 10.0)	1.9 (0.5, 4.0)

E1: With endocapillary proliferation, E0: Without endocapillary proliferation.

* indicates p<0.05 for difference between E1 and E0. Data are presented as mean (SD) unless otherwise indicated.

### Glomerular Transcriptome analysis

We identified 424 genes that are differentially expressed in microdissected glomeruli with endocapillary proliferation versus glomeruli without endocapillary proliferation at a conservative false-discovery-adjusted q-value of <0.05 (Table 2, full list Table S2). The expression of a subset of these transcripts also correlated with renal function (estimated glomerular filtration rate) at the time of biopsy (Table 2). These transcripts demonstrated an inverse correlation with renal function, and the top candidates include transcripts encoding proteins associated with biologic processes including innate immune response and classical pathway complement activation (C1QA, C1QB, C2, VSIG4), and matrix degradation and turnover (HSPE, TIMP1). In addition, CD163, a marker for M2 macrophage polarization associated with IgA nephropathy in adults, was highly upregulated and negatively correlated with renal function. We also evaluated the correlation between mRNA expression and endocapillary expression, considering endocapillary hypercellularity as a continuous variable and confirmed that the expression of a subset of these transcripts also correlates with the severity of endocapillary proliferation (Figure S1, Table S3).

**Table 2:** Correlation of differentially expressed transcripts with renal function.

		Differential expression E1 vs. E0		Correlation of expression with eGFR	
Gene symbol	Gene name	Fold-change	q-value	r	FDR
AURKB	Aurora kinase B	1.8	0.02	−0.66	0.03
C1QA	Complement component 1, q sub component, A chain	4.11	0.01	−0.69	0.03
C1QB	Complement component 1, q sub component, B chain	6.04	0.01	−0.75	0.01
C2	Complement component 2	1.5	0.04	−0.66	0.03
CD163	CD163 molecule	6.28	0.00	−0.63	0.04
CD44	CD44 molecule (Indian blood group)	2.04	0.02	−0.68	0.03
CORO1C	Coronin, actin binding protein, 1C	1.7	0.01	−0.64	0.04
FOXM1	Forkhead box M1	2.33	0.03	−0.63	0.04
GPR183	G protein-coupled receptor 183	2.59	0.04	−0.69	0.03
HPSE	Heparanase	2.34	0.01	−0.75	0.01
KIF15	Kinesin family member 15	1.93	0.02	−0.65	0.04
LAIR1	Leukocyte-associated immunoglobulin-like receptor 1	2.16	0.01	−0.71	0.02
LAPTM5	Lysosomal protein transmembrane 5	2.27	0.01	−0.68	0.03
LHFPL2	Lipoma HMGIC fusion partner-like 2	1.98	0.01	−0.71	0.02
MAN2B1	Mannosidase, alpha, class 2B, member 1	1.48	0.04	−0.64	0.04
MERTK	c-merproto-oncogene tyrosine kinase	1.43	0.04	−0.66	0.03
MS4A6A	Membrane-spanning 4-domains, subfamily A, member 6A	2.34	0.01	−0.64	0.04
MS4A6A	Membrane-spanning 4-domains, subfamily A, member 6A	2.34	0.01	−0.69	0.03
PRC1	Protein regulator of cytokinesis 1	2.61	0.04	−0.63	0.04
SLC43A3	Solute carrier family 43, member 3	1.74	0.01	−0.63	0.04
TIMP1	TIMP metallopeptidase inhibitor 1	1.86	0.03	−0.70	0.03
VSIG4	V-set and immunoglobulin domain containing 4	5.5	0.00	−0.68	0.03

Pearson correlation coefficients (r) are shown for the relationship between mRNA expression and GFR estimated using the CKD-Epi equation. Transcripts in this table were chosen based on false discovery rate (FDR) <0.05.

**Figure 1:**
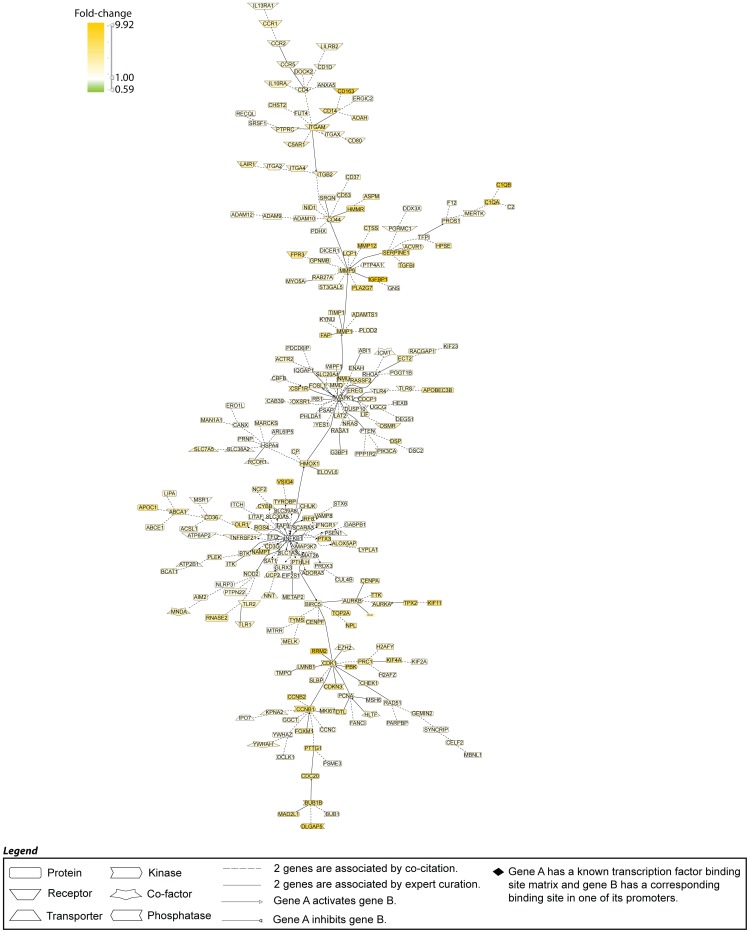
Literature-based analysis from the 424 genes regulated in E1 vs. E0 biopsies using Genomatix Pathway System (GePS) software. The picture shows the 248 genes that were co-cited in PubMed abstracts in the same sentence with a function-word filter.

To identify relevant biological pathways represented in these 424 differentially expressed genes, we performed canonical pathway analysis (Ingenuity Pathway Analysis). The top enriched canonical pathways differentially expressed in biopsies with endocapillary proliferation are provided in Table 3 (full list Table S4). The predominant biologic processes represented in this analysis included innate immune response (ex. toll-like receptors), inflammatory, and T-cell signaling pathways. In addition, transcripts encoding regulators of cell cycle and division were overrepresented in the differentially expressed transcripts. Literature-based analysis of enriched biologic pathways is illustrated in Figure 1.

**Table 3:** Canonical pathway analysis.

Canonical pathways (number of genes regulated/number of genes in the pathway)	p-value	Regulated molecules in the pathway
**Role of Pattern Recognition Receptors in Recognition of Bacteria and Viruses (14/106)**	0.0000	PTX3,TLR1,PIK3CA,NLRP3,MAPK1,TLR8, C1QA,C1QB,EIF2S1,TLR2,TLR4,NOD2, C5AR1, PRKD3
**Leukocyte Extravasation Signaling (18/201)**	0.0001	PIK3CA,MAPK1,ITGA2,BTK,ITGB2,WIPF1, ITGAM,TIMP1,RHOA,NCF2,CD44,CYBB, MMP12,PRKD3,MMP9,MMP1,ITGA4,ITK
**TREM1 Signaling (9/71)**	0.0001	TLR2,TLR4,TLR1,NOD2,MAPK1,TYROBP, TLR8,LAT2,ITGAX
**NF-κB Activation by Viruses (10/82)**	0.0003	ITGB2,PIK3CA,CCR5,NRAS,MAPK1,CD4, ITGA2,CHUK,PRKD3,ITGA4
**Toll-like Receptor Signaling (8/62)**	0.0004	TLR2,TLR4,TLR1,MAPK1,MAP3K7,TLR8, CD14,CHUK
**Cell Cycle: G2/M DNA Damage Checkpoint Regulation (7/48)**	0.0006	YWHAH,YWHAZ,TOP2A,CCNB2,CDK1, CHEK1,CCNB1
**IL-8 Signaling (16/205)**	0.0006	PIK3CA,NRAS,MAPK1,IQGAP1,RAB11FIP2, ITGB2,HMOX1,ITGAM,RHOA,NCF2,CYBB, CHUK,PRKD3,GNG12,MMP9,ITGAX
**Production of Nitric Oxide and Reactive Oxygen Species in Macrophages (13/210)**	0.0089	PIK3CA,MAPK1,IFNGR1,TLR2,APOC1,TLR4, MAP3K7,RHOA,NCF2,CYBB,IRF8,CHUK, PRKD3

Selected canonical pathways significantly regulated (p-value<0.05) from the 424 genes regulated in E1 vs. E0 biopsies, as assessed by Ingenuity Pathway Analysis. Full pathway list in Table S3.

The only glomerular pathologic phenotype as defined by the Oxford classification associated with a distinct molecular signature was endocapillary hypercellularity. Only 9 and 4 genes were significantly regulated in biopsies with versus without mesangial hypercellularity or segmental glomerulosclerosis, respectively.

### Links to corticosteroid therapy: NFκB-regulated transcripts

Therapy with corticosteroid is associated with complete regression of endocapillary proliferation in serial biopsies of patients with IgAN. Given that the primary mechanism of action underlying the immunosuppressive action of prednisone is interruption of transcription of nuclear factor kappa-B-(NFκB) regulated genes, we performed transcription factor analysis to evaluate whether there was an overrepresentation of genes with an upstream NFκB consensus binding unit. We found that one quarter (144 of 424) of the differentially expressed genes contained upstream NFκB promoter sites representing significant enrichment of pathways that may be modulated by inhibition of corticosteroids (Table S5). Using a list of all the human transcription factors, TP53 and NFKB1 were the two top transcription factor having a binding site in the promoter of the E1 vs. E0 regulated genes. The full results of transcription factor analysis are provided in Table S6.

### Connectivity mapping and drug pairing prediction: in silico drug screening

To identify bioactive compounds that modulate expression of the transcripts represented in our endocapillary proliferation signature, we used the Connectivity Map (CMAP) approach. With this approach we compared our gene signatures to expression profiles in experimental *in vitro* models of cellular responses to a panel of bioactive molecules [27]. Using this unbiased methodology we confirmed that the compounds predicted in silico to reverse gene expression changes associated with endocapillary proliferation included methylprednisolone (107 genes) and corticosterone (85 genes) (adjusted p<0.05). With this approach, we also identified a set of bioactive compounds that would be predicted to have favorable biologic activity to reverse the transcriptional responses associated with endocapillary proliferation, including molecules previously used to treat other forms of glomerulonephritis (hydroquinine, ciclosporin and methotrexate) and several novel compounds not previously studied in endocapillary-proliferation within IgAN, such as resveratrol (Table 4, full list in Table S7). The Drug Pair Seeker program was used to predict which drug would enhance the reversal gene expression in endocapillary proliferation when combined with methylprednisolone or corticosterone treatment. With this analysis tool, resveratrol was predicted to have potentially beneficial effects when used in combination with corticosteroid-based regimens (p<0.05, Table S8).

**Table 4:** Selected compounds of interest from Connectivity map analysis results (p-value<0.05).

Rank	Cmap name	p
1	Hydroquinine	0.0002
3	Resveratrol	0.0003
8	guaifenesin	0.0016
10	methotrexate	0.0025
12	genistein	0.0061
24	ciclosporin	0.0148
64	corticosterone	0.0324
72	methylprednisolone	0.0405

These are bioactive compounds that would be predicted to have favorable biologic activity to modulate the transcriptional responses associated with endocapillary proliferation. Full results of the analysis are available in Table S6.

## Discussion

We have employed a translational approach to delineate molecular mechanisms underlying the development of endocapillary proliferation in human subjects with IgAN, and to identify potential therapeutic targets to modulate this disease. Our approach to identification of clinically and biologically relevant processes involved in progressive IgAN was based upon two important clinical observations. First, the pathologic lesion of endocapillary proliferation is associated with a higher risk of progressive loss of renal function, and second, it is potentially modifiable by corticosteroid therapy.

Large observational studies suggest that corticosteroids may be effective in preventing progression of IgAN [22]. However, corticosteroids have broad cellular and biologic effects with significant toxicity. Identification of the specific molecules and pathways upregulated in endocapillary proliferation and potentially modulated by corticosteroids may yield information for development of more targeted therapy focused on the downstream genes and proteins regulated by NFκB.

Our first finding is that in the isolated microdissected glomeruli of human diagnostic kidney biopsies, endocapillary proliferation is associated with a distinct signature of differentially expressed mRNA transcripts. We identified the biologic processes and pathways represented in this signature, using canonical pathway analysis. Given that endocapillary proliferation may be amenable to therapy with corticosteroids and that the prime mechanism of action of this drug is interruption of NFκB-mediated gene transcription, we verified that our signature includes transcripts regulated by this transcription factor. Indeed one-third of the transcripts contain upstream promoter binding sites. Finally, we employed in silico drug screening and confirmed that the endocapillary proliferation transcriptome is significantly enriched with pathways modulated by corticosteroids, which is supportive of clinical observations of efficacy of corticosteroids in patients with this lesion. With this approach we also identified novel therapeutic targets and bioactive small molecules that may be considered for treatment of IgAN.

The glomerular mRNA expression profile associated with endocapillary proliferation includes a significant number of transcripts encoding proteins involved in the innate immune response, such as several toll-like receptors (TLRs). One of the potential mechanisms hypothesized to be responsible for development of IgAN is a dysregulated immunologic response to a mucosal microbial challenge [23], [24]. The innate immune system is a first line of rapid defense against foreign microbial invasion at the mucosal surface, and TLRs are central in the process of activation of the innate immune response following recognition of specific patterns of microbial components [25]. Our findings of increased TLR expression support activation of the innate immune response in development of endocapillary proliferation, and a next step would be to identify whether the expression is from infiltrating versus resident cells. In addition to expression by monocytes/macrophages, and dendritic cells, TLR expression has been demonstrated by renal endothelial cells, mesangial cells and podocytes (reviewed in [26]).

The activation of TLRs and binding of intracellular adapter proteins results in increased activity of transcription factors interferon transcription factor 3 and NFκB [27]. It is tempting to speculate that one of the mechanisms by which corticosteroids are beneficial in this lesion is by blockade of the downstream activation of TLRs. More specific approaches to blockade of TLR activation and signaling may therefore offer more targeted therapy in patients with this high risk lesion.

To evaluate functional connections between corticosteroids and our differentially expressed transcripts we used CMAP analysis. This tool is a reference catalogue of gene expression data derived from cultured cells exposed to a spectrum of bioactive compounds [20]. We sought to predict if our differentially expressed transcripts demonstrates potential favorable response to a panel of compounds tested in a variety of in vitro cell systems. This novel systems pharmacology tool has recently been shown to identify drug targets for repurposing in rare diseases [28] and has recently been applied to prediction of drug combination efficacy in experimental murine models of kidney disease [29]. Drugs predicted in silico to favorably alter the profile of differentially expressed genes in HIV-associated nephropathy were indeed protective when tested in vivo. While this strategy of drug discovery is in early stages, tools such as CMAP facilitate evaluation of transcriptome data and identification of potential therapeutic pathways and have not yet been applied to studies of glomerulonephritis. The CMAP analysis has been integrated by us into the systems biology tool suite in Nephromine and can be readily employed by the scientific community not only to IgAN but to all glomerular diseases available in Nephromine (www.Nephropmine.org). The Connectivity Map datasets have been used to predict and prioritize pairs of drugs that reverse or aggravate the direction of differential gene expression (“Drug Pair Seeker” available at http://www.maayanlab.net/DPS/). With this approach we identified several compounds predicted to reverse the gene expression changes associated with endocapillary proliferation. Resveretrol emerged as a potential novel compound with this potential therapeutic effect, and interestingly in our analysis both methylprednisolone and corticosterone were predicted to reverse the E1 transcriptional responses when used in combination with resveratrol. This compound, a polyphenol naturally occurring in grapes, has been shown to have attenuate kidney injury induced in the ischemia-reperfusion rat model [30] through stimulation of nitric oxide production and moderation of oxidative stress. Most recently, resveratrol has been found to attenuate experimental diabetic nephropathy, related in part to suppression of VEGF and angiopoietin 2 -induced changes in glomerular permeability [31]. The favourable tolerability profile of this compound in humans make this a potential new agent for therapeutic trial [32].

In summary, we have used a novel approach to identify a distinct glomerular molecular signature associated with endocapillary proliferation. Our transcriptional and pathway analyses support the clinical observation that this lesion may be modifiable with use of corticosteroids. We have also identified therapeutic targets and agents for future study in treatment of IgAN.

## Supporting Information

Figure S1 **Analytic approach.**
(DOCX)

Table S1 **Oxford MEST scoring.**
(DOCX)

Table S2 **List of the 424 genes significantly differentially regulated in E1 vs. E0 IgAN biopsies (q-value<0.05), sorted by q-value.** The 144 genes in **bold type** also possess an upstream binding site for NFκB1 in their promoter region.
(DOCX)

Table S3 **Considering endocapillary proliferation as a continuous variable.** As illustrated in Figure S1, we also correlated mRNA expression with endocapillary proliferation severity (ie. endocapillary proliferation considered as a continuous variable). With this approach we identified 141 transcripts that are: a – differentially expressed in E1 vs. E0 biopsies and b – correlated with the the relative degree of endocapillary proliferation.
(DOCX)

Table S4 **Canonical pathways significantly regulated (p-value<0.05) from the 424 genes regulated in E1 vs. E0 biopsies, as assessed by IPA (Ingenuity Pathway Analysis).**
(DOCX)

Table S5 **144 genes of the 424 regulated in E1 vs E0 have a binding site for NFKB1 in their promoter.**
(DOCX)

Table S6 **Transcription factor analysis results.** This list includes TFs able to bind to the promoter region of at least 30 different genes from the 424 genes differentially regulated in E1 vs E0.
(DOCX)

Table S7 **Compounds from Connectivity map analysis results (p-value<0.05).** These compounds are predicted to have favourable effects reversing the majority of differential mRNA expression changes associated with endocapillary proliferation. Compounds of interest are highlighted in grey.
(DOCX)

Table S8 **Drug pair seeker analysis.** Top computationally-predicted drugs that would enhance the reversal of gene expression changes associated with endocapillary proliferation when combined with methylprednisolone or corticosterone. Coverage = number of desirable targets that the drug affects, meaning the transcripts described in endocapillary proliferation that the drug would reverse. Conflict = the number of genes the drug is potentially changing in an undesired direction in endocapillary proliferation.
(DOCX)
