# Supplementary figures and images for: The Molecular Phenotype of Endocapillary Proliferation: Novel Therapeutic Targets for IgA Nephropathy
Source: PLoS One. 2014 Aug 18;9(8):e103413. doi: 10.1371/journal.pone.0103413 (PMC4136785; doi:10.1371/journal.pone.0103413)

Supplementary Figure S1. Analytic approach.


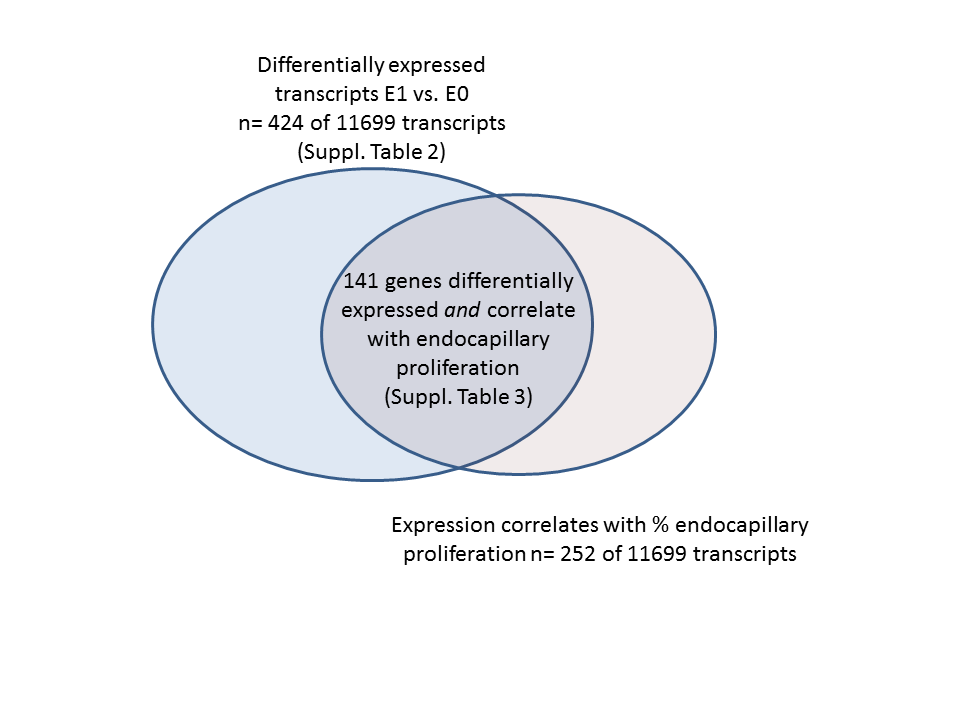

Supplement: Figure S1 — Analytic approach. (DOCX) [file pone.0103413.s001.docx]
