# Supplementary material for: The Molecular Phenotype of Endocapillary Proliferation: Novel Therapeutic Targets for IgA Nephropathy
Source: PLoS One. 2014 Aug 18;9(8):e103413. doi: 10.1371/journal.pone.0103413 (PMC4136785; doi:10.1371/journal.pone.0103413)
Supplement: Table S1 — Oxford MEST scoring. (DOCX) [file pone.0103413.s002.docx]

**Supplementary Table S1: Oxford MEST scoring.** Adapted from Roberts et al. Kidney Int. 2009 76(5): 546.

**Mesangial hypercellularity**

M0: Score <0.5

M1: Score >0.5

**Endocapillary proliferation**

E0: Absent

E1: Present

**Segmental glomerulosclerosis**

S0: Absent

S1: Present

**Tubular atrophy/interstitial fibrosis**

T0: 0-25% of cortical area

T1: 26-50% of cortical area

T2: >50% of cortical area
