# Supplementary material for: The Molecular Phenotype of Endocapillary Proliferation: Novel Therapeutic Targets for IgA Nephropathy
Source: PLoS One. 2014 Aug 18;9(8):e103413. doi: 10.1371/journal.pone.0103413 (PMC4136785; doi:10.1371/journal.pone.0103413)
Supplement: Table S2 — List of the 424 genes significantly differentially regulated in E1 vs. E0 IgAN biopsies (q-value<0.05), sorted by q-value. The 144 genes in bold type also possess an upstream binding site for NFκB1 in their promoter region. (DOCX) [file pone.0103413.s003.docx]

**Supplementary Table S2.** List of the 424 genes significantly differentially regulated in E1 vs. E0 IgAN biopsies (q-value<0.05), sorted by q-value. The 144 genes in **bold type** also possess an upstream binding site for NFκB1 in their promoter region.

| Entrez Gene ID | Gene Symbol | Fold-change | q-value |
| --- | --- | --- | --- |
| 55608 | ANKRD10 | 1.53 | 0.0000 |
| 6059 | ABCE1 | 1.60 | 0.0000 |
| 22974 | TPX2 | 3.47 | 0.0000 |
| 81537 | SGPP1 | 1.69 | 0.0000 |
| 3832 | KIF11 | 3.85 | 0.0000 |
| **9787** | **DLGAP5** | **4.80** | **0.0000** |
| 51338 | MS4A4A | 5.38 | 0.0000 |
| 1058 | CENPA | 2.20 | 0.0000 |
| 51514 | DTL | 2.69 | 0.0000 |
| 7045 | TGFBI | 2.46 | 0.0000 |
| 701 | BUB1B | 3.61 | 0.0000 |
| **83737** | **ITCH** | **1.35** | **0.0000** |
| **6241** | **RRM2** | **6.92** | **0.0000** |
| 6745 | SSR1 | 1.35 | 0.0000 |
| 7272 | TTK | 2.58 | 0.0000 |
| 4200 | ME2 | 1.76 | 0.0000 |
| 10112 | KIF20A | 2.26 | 0.0000 |
| 8522 | GAS7 | 1.50 | 0.0000 |
| **9232** | **PTTG1** | **3.07** | **0.0000** |
| **5888** | **RAD51** | **1.42** | **0.0000** |
| **1033** | **CDKN3** | **2.94** | **0.0000** |
| **5054** | **SERPINE1** | **3.29** | **0.0000** |
| 51133 | KCTD3 | 1.72 | 0.0000 |
| 11326 | VSIG4 | 5.50 | 0.0000 |
| 58504 | ARHGAP22 | 2.17 | 0.0000 |
| 6039 | RNASE6 | 3.53 | 0.0000 |
| **10135** | **NAMPT** | **2.78** | **0.0000** |
| 51809 | GALNT7 | 2.33 | 0.0000 |
| **9332** | **CD163** | **6.28** | **0.0000** |
| 55197 | RPRD1A | 1.50 | 0.0000 |
| 80183 | C13orf18 | 2.59 | 0.0000 |
| **4644** | **MYO5A** | **1.60** | **0.0000** |
| 55159 | RFWD3 | 1.91 | 0.0000 |
| **3684** | **ITGAM** | **2.67** | **0.0000** |
| 51571 | FAM49B | 1.87 | 0.0000 |
| **10097** | **ACTR2** | **1.64** | **0.0000** |
| 4085 | MAD2L1 | 2.68 | 0.0000 |
| 1230 | CCR1 | 1.94 | 0.0000 |
| 9555 | H2AFY | 1.75 | 0.0000 |
| 259266 | ASPM | 2.10 | 0.0000 |
| 81542 | TMX1 | 1.61 | 0.0000 |
| 10434 | LYPLA1 | 1.67 | 0.0000 |
| 55165 | CEP55 | 4.62 | 0.0000 |
| **90** | **ACVR1** | **1.87** | **0.0000** |
| 55355 | HJURP | 1.83 | 0.0000 |
| 51714 | SELT | 1.80 | 0.0000 |
| **55872** | **PBK** | **4.84** | **0.0000** |
| 3161 | HMMR | 3.15 | 0.0000 |
| **1536** | **CYBB** | **2.31** | **0.0000** |
| 51167 | CYB5R4 | 2.37 | 0.0000 |
| 10228 | STX6 | 1.29 | 0.0000 |
| 2359 | FPR3 | 3.13 | 0.0000 |
| **1111** | **CHEK1** | **1.72** | **0.0000** |
| 10973 | ASCC3 | 1.72 | 0.0000 |
| **19** | **ABCA1** | **2.27** | **0.0000** |
| **3484** | **IGFBP1** | **9.89** | **0.0000** |
| **3676** | **ITGA4** | **1.80** | **0.0000** |
| 29127 | RACGAP1 | 1.63 | 0.0067 |
| 10184 | LHFPL2 | 1.98 | 0.0067 |
| 9133 | CCNB2 | 3.43 | 0.0067 |
| 55970 | GNG12 | 1.40 | 0.0067 |
| **8560** | **DEGS1** | **1.30** | **0.0070** |
| 729230 | CCR2 | 1.60 | 0.0070 |
| **3394** | **IRF8** | **2.42** | **0.0070** |
| 23484 | LEPROTL1 | 1.38 | 0.0070 |
| 699 | BUB1 | 1.46 | 0.0070 |
| 10564 | ARFGEF2 | 1.40 | 0.0070 |
| 51719 | CAB39 | 1.43 | 0.0070 |
| 64151 | NCAPG | 2.52 | 0.0070 |
| 56833 | SLAMF8 | 1.75 | 0.0070 |
| 29015 | SLC43A3 | 1.74 | 0.0070 |
| **10146** | **G3BP1** | **1.43** | **0.0070** |
| 9493 | KIF23 | 1.40 | 0.0070 |
| 23471 | TRAM1 | 1.31 | 0.0070 |
| **5621** | **PRNP** | **1.36** | **0.0070** |
| **891** | **CCNB1** | **3.50** | **0.0070** |
| 7941 | PLA2G7 | 3.50 | 0.0082 |
| 2799 | GNS | 1.30 | 0.0083 |
| 713 | C1QB | 6.04 | 0.0084 |
| **3903** | **LAIR1** | **2.16** | **0.0085** |
| 10457 | GPNMB | 1.83 | 0.0085 |
| 79682 | MLF1IP | 2.19 | 0.0087 |
| 313 | AOAH | 1.99 | 0.0088 |
| **7534** | **YWHAZ** | **1.29** | **0.0088** |
| **1436** | **CSF1R** | **2.38** | **0.0088** |
| 2124 | EVI2B | 3.59 | 0.0092 |
| 7803 | PTP4A1 | 1.37 | 0.0092 |
| 51368 | TEX264 | 0.59 | 0.0094 |
| 3590 | IL11RA | 0.65 | 0.0094 |
| 4288 | MKI67 | 1.76 | 0.0097 |
| 55706 | TMEM48 | 1.39 | 0.0098 |
| 1520 | CTSS | 2.00 | 0.0098 |
| 55379 | LRRC59 | 1.39 | 0.0098 |
| 8477 | GPR65 | 2.24 | 0.0098 |
| **10855** | **HPSE** | **2.34** | **0.0098** |
| **9510** | **ADAMTS1** | **1.89** | **0.0098** |
| **9770** | **RASSF2** | **2.20** | **0.0098** |
| 8450 | CUL4B | 1.36 | 0.0098 |
| 9582 | APOBEC3B | 2.65 | 0.0098 |
| 6856 | SYPL1 | 1.29 | 0.0098 |
| 51203 | NUSAP1 | 2.84 | 0.0098 |
| 79709 | GLT25D1 | 1.52 | 0.0098 |
| **712** | **C1QA** | **4.11** | **0.0098** |
| 1894 | ECT2 | 2.01 | 0.0098 |
| **3071** | **NCKAP1L** | **1.89** | **0.0098** |
| 5873 | RAB27A | 1.60 | 0.0098 |
| 10006 | ABI1 | 1.32 | 0.0098 |
| **341** | **APOC1** | **3.24** | **0.0098** |
| 8540 | AGPS | 1.73 | 0.0098 |
| 10857 | PGRMC1 | 1.37 | 0.0103 |
| 7456 | WIPF1 | 1.48 | 0.0104 |
| **8754** | **ADAM9** | **1.54** | **0.0104** |
| 25939 | SAMHD1 | 1.55 | 0.0104 |
| 51582 | AZIN1 | 1.46 | 0.0104 |
| 54478 | FAM64A | 1.93 | 0.0104 |
| 4321 | MMP12 | 4.04 | 0.0104 |
| 5917 | RARS | 1.36 | 0.0108 |
| 79666 | PLEKHF2 | 1.66 | 0.0108 |
| 23186 | RCOR1 | 1.53 | 0.0108 |
| **728** | **C5AR1** | **2.11** | **0.0108** |
| **79718** | **TBL1XR1** | **1.48** | **0.0112** |
| **5229** | **PGGT1B** | **1.36** | **0.0112** |
| 10613 | ERLIN1 | 1.31 | 0.0112 |
| 7884 | SLBP | 1.73 | 0.0112 |
| 2589 | GALNT1 | 1.43 | 0.0112 |
| **8869** | **ST3GAL5** | **1.62** | **0.0112** |
| 57405 | SPC25 | 1.70 | 0.0112 |
| 54149 | C21orf91 | 1.35 | 0.0112 |
| 81553 | FAM49A | 1.55 | 0.0112 |
| 64231 | MS4A6A | 2.34 | 0.0112 |
| **821** | **CANX** | **1.31** | **0.0112** |
| 55010 | C12orf48 | 1.39 | 0.0112 |
| **29028** | **ATAD2** | **1.68** | **0.0112** |
| 10484 | SEC23A | 1.54 | 0.0112 |
| **4001** | **LMNB1** | **2.00** | **0.0112** |
| 2123 | EVI2A | 2.50 | 0.0124 |
| 2161 | F12 | 1.43 | 0.0125 |
| 6790 | AURKA | 1.93 | 0.0125 |
| 23603 | CORO1C | 1.70 | 0.0125 |
| 51762 | RAB8B | 1.32 | 0.0125 |
| 6574 | SLC20A1 | 1.78 | 0.0126 |
| 586 | BCAT1 | 1.55 | 0.0126 |
| **64924** | **SLC30A5** | **1.43** | **0.0129** |
| **5806** | **PTX3** | **3.73** | **0.0129** |
| **917** | **CD3G** | **1.61** | **0.0136** |
| 64866 | CDCP1 | 1.74 | 0.0137 |
| **5111** | **PCNA** | **1.50** | **0.0137** |
| **892** | **CCNC** | **1.40** | **0.0137** |
| 7153 | TOP2A | 3.18 | 0.0137 |
| **5627** | **PROS1** | **1.54** | **0.0140** |
| **5594** | **MAPK1** | **1.44** | **0.0141** |
| **7805** | **LAPTM5** | **2.27** | **0.0141** |
| **1794** | **DOCK2** | **1.72** | **0.0143** |
| **2526** | **FUT4** | **1.38** | **0.0143** |
| 9375 | TM9SF2 | 1.35 | 0.0143 |
| 29929 | ALG6 | 1.57 | 0.0146 |
| **64092** | **SAMSN1** | **2.46** | **0.0146** |
| 10565 | ARFGEF1 | 1.39 | 0.0146 |
| **387** | **RHOA** | **1.23** | **0.0170** |
| **5788** | **PTPRC** | **1.73** | **0.0170** |
| 6426 | SFRS1 | 1.33 | 0.0170 |
| **24137** | **KIF4A** | **2.84** | **0.0173** |
| **4312** | **MMP1** | **2.54** | **0.0174** |
| 5352 | PLOD2 | 1.57 | 0.0175 |
| **6885** | **MAP3K7** | **1.45** | **0.0175** |
| 10981 | RAB32 | 1.44 | 0.0175 |
| 7326 | UBE2G1 | 1.39 | 0.0175 |
| 54491 | FAM105A | 1.29 | 0.0175 |
| 8140 | SLC7A5 | 1.96 | 0.0186 |
| **7112** | **TMPO** | **1.54** | **0.0187** |
| **25816** | **TNFAIP8** | **1.79** | **0.0188** |
| 1063 | CENPF | 2.11 | 0.0188 |
| **960** | **CD44** | **2.04** | **0.0190** |
| 26191 | PTPN22 | 1.35 | 0.0194 |
| **920** | **CD4** | **1.56** | **0.0194** |
| 92342 | C1orf156 | 1.42 | 0.0196 |
| 6596 | HLTF | 1.55 | 0.0197 |
| 11339 | OIP5 | 1.51 | 0.0197 |
| 54107 | POLE3 | 1.61 | 0.0200 |
| 9928 | KIF14 | 1.37 | 0.0200 |
| 8406 | SRPX | 1.72 | 0.0202 |
| 8487 | SIP1 | 1.51 | 0.0202 |
| 4811 | NID1 | 1.93 | 0.0202 |
| 9180 | OSMR | 1.90 | 0.0202 |
| 23244 | PDS5A | 1.33 | 0.0211 |
| 2191 | FAP | 3.34 | 0.0211 |
| 3936 | LCP1 | 2.30 | 0.0211 |
| 79017 | GGCT | 1.39 | 0.0211 |
| 10403 | NDC80 | 1.98 | 0.0211 |
| **5290** | **PIK3CA** | **1.49** | **0.0211** |
| 28966 | SNX24 | 1.36 | 0.0218 |
| 3074 | HEXB | 1.34 | 0.0218 |
| 983 | CDC2 | 2.68 | 0.0218 |
| **2146** | **EZH2** | **1.59** | **0.0218** |
| 56992 | KIF15 | 1.93 | 0.0218 |
| 6643 | SNX2 | 1.22 | 0.0218 |
| 9943 | OXSR1 | 1.60 | 0.0218 |
| 9326 | ZNHIT3 | 1.33 | 0.0223 |
| 117178 | SSX2IP | 1.38 | 0.0223 |
| 9212 | AURKB | 1.80 | 0.0223 |
| 8761 | PABPC4 | 1.49 | 0.0226 |
| 4082 | MARCKS | 1.52 | 0.0227 |
| **5921** | **RASA1** | **1.45** | **0.0227** |
| 8914 | TIMELESS | 1.19 | 0.0227 |
| 64747 | MFSD1 | 1.36 | 0.0227 |
| **64127** | **NOD2** | **1.47** | **0.0229** |
| 23683 | PRKD3 | 1.37 | 0.0229 |
| 3015 | H2AFZ | 1.44 | 0.0232 |
| 81555 | YIPF5 | 1.23 | 0.0237 |
| 10492 | SYNCRIP | 1.33 | 0.0238 |
| 11052 | CPSF6 | 1.31 | 0.0239 |
| **8038** | **ADAM12** | **1.42** | **0.0250** |
| 23405 | DICER1 | 1.27 | 0.0250 |
| **1965** | **EIF2S1** | **1.26** | **0.0250** |
| **23531** | **MMD** | **1.65** | **0.0250** |
| **8061** | **FOSL1** | **1.68** | **0.0252** |
| **7096** | **TLR1** | **1.79** | **0.0252** |
| 26135 | SERBP1 | 1.25 | 0.0252 |
| **57018** | **CCNL1** | **1.42** | **0.0252** |
| 55435 | C4orf16 | 1.64 | 0.0252 |
| 55619 | DOCK10 | 2.21 | 0.0252 |
| 23167 | EFR3A | 1.28 | 0.0252 |
| 11010 | GLIPR1 | 1.90 | 0.0252 |
| 23042 | PDXDC1 | 1.32 | 0.0253 |
| 80896 | NPL | 3.21 | 0.0253 |
| 8050 | PDHX | 1.35 | 0.0253 |
| **54407** | **SLC38A2** | **1.36** | **0.0253** |
| **2069** | **EREG** | **1.65** | **0.0253** |
| 9050 | PSTPIP2 | 1.48 | 0.0253 |
| **5925** | **RB1** | **1.37** | **0.0253** |
| **2956** | **MSH6** | **1.25** | **0.0253** |
| 10527 | IPO7 | 1.27 | 0.0253 |
| 4688 | NCF2 | 1.82 | 0.0253 |
| 7323 | UBE2D3 | 1.32 | 0.0253 |
| **1234** | **CCR5** | **1.65** | **0.0253** |
| 526 | ATP6V1B2 | 1.42 | 0.0253 |
| **7533** | **YWHAH** | **1.63** | **0.0253** |
| 10988 | METAP2 | 1.38 | 0.0253 |
| 22822 | PHLDA1 | 1.31 | 0.0259 |
| **22797** | **TFEC** | **2.07** | **0.0259** |
| **4481** | **MSR1** | **1.25** | **0.0261** |
| 51444 | RNF138 | 1.51 | 0.0261 |
| 178 | AGL | 1.37 | 0.0264 |
| 929 | CD14 | 2.63 | 0.0264 |
| 9201 | DCLK1 | 1.28 | 0.0264 |
| 23530 | NNT | 1.70 | 0.0264 |
| **102** | **ADAM10** | **1.52** | **0.0264** |
| 991 | CDC20 | 2.69 | 0.0264 |
| 515 | ATP5F1 | 1.26 | 0.0264 |
| 81533 | ITFG1 | 1.43 | 0.0264 |
| 10051 | SMC4 | 1.51 | 0.0264 |
| 10737 | RFPL3S | 1.34 | 0.0264 |
| 29902 | C12orf24 | 1.44 | 0.0264 |
| **1824** | **DSC2** | **1.31** | **0.0264** |
| **3587** | **IL10RA** | **1.81** | **0.0264** |
| 7357 | UGCG | 1.42 | 0.0264 |
| 55273 | TMEM100 | 2.05 | 0.0264 |
| 55233 | MOBKL1B | 1.58 | 0.0269 |
| 830 | CAPZA2 | 1.34 | 0.0269 |
| 253943 | YTHDF3 | 1.38 | 0.0269 |
| 54877 | ZCCHC2 | 1.56 | 0.0272 |
| 963 | CD53 | 1.84 | 0.0272 |
| **2305** | **FOXM1** | **2.33** | **0.0272** |
| **64116** | **SLC39A8** | **1.38** | **0.0272** |
| 55156 | ARMC1 | 1.45 | 0.0272 |
| 9926 | LPGAT1 | 1.55 | 0.0272 |
| 56938 | ARNTL2 | 1.59 | 0.0272 |
| 29957 | SLC25A24 | 1.40 | 0.0272 |
| 54928 | IMPAD1 | 1.30 | 0.0272 |
| **4144** | **MAT2A** | **1.32** | **0.0275** |
| 5504 | PPP1R2 | 1.41 | 0.0275 |
| 81578 | COL21A1 | 1.41 | 0.0275 |
| 8942 | KYNU | 1.38 | 0.0275 |
| 2585 | GALK2 | 1.36 | 0.0275 |
| **3702** | **ITK** | **1.49** | **0.0275** |
| 79624 | C6orf211 | 1.69 | 0.0275 |
| 490 | ATP2B1 | 1.31 | 0.0299 |
| 26235 | FBXL4 | 1.48 | 0.0300 |
| **7525** | **YES1** | **1.38** | **0.0300** |
| **2180** | **ACSL1** | **1.56** | **0.0300** |
| **7076** | **TIMP1** | **1.86** | **0.0303** |
| 4154 | MBNL1 | 1.30 | 0.0303 |
| **332** | **BIRC5** | **1.66** | **0.0303** |
| **5663** | **PSEN1** | **1.24** | **0.0303** |
| 3059 | HCLS1 | 1.70 | 0.0303 |
| **27242** | **TNFRSF21** | **1.56** | **0.0303** |
| 3988 | LIPA | 1.51 | 0.0303 |
| 51727 | CMPK1 | 1.40 | 0.0307 |
| 3612 | IMPA1 | 1.41 | 0.0307 |
| **3673** | **ITGA2** | **1.52** | **0.0307** |
| **6507** | **SLC1A3** | **1.41** | **0.0307** |
| 63933 | CCDC90A | 1.54 | 0.0318 |
| 10959 | TMED2 | 1.34 | 0.0326 |
| 4332 | MNDA | 1.87 | 0.0326 |
| 5999 | RGS4 | 2.20 | 0.0326 |
| **7298** | **TYMS** | **2.18** | **0.0326** |
| **7305** | **TYROBP** | **1.97** | **0.0329** |
| **10539** | **GLRX3** | **1.30** | **0.0329** |
| **9447** | **AIM2** | **1.50** | **0.0329** |
| **695** | **BTK** | **1.61** | **0.0329** |
| 22948 | CCT5 | 1.45 | 0.0329 |
| 10479 | SLC9A6 | 1.32 | 0.0329 |
| 9889 | ZBED4 | 1.39 | 0.0329 |
| **3976** | **LIF** | **1.62** | **0.0330** |
| 22841 | RAB11FIP2 | 1.32 | 0.0330 |
| 4552 | MTRR | 1.27 | 0.0330 |
| 5965 | RECQL | 1.30 | 0.0333 |
| **10935** | **PRDX3** | **1.32** | **0.0333** |
| 55198 | APPL2 | 1.43 | 0.0333 |
| 10874 | NMU | 2.88 | 0.0333 |
| 11130 | ZWINT | 1.68 | 0.0333 |
| 55699 | IARS2 | 1.21 | 0.0333 |
| 10197 | PSME3 | 1.20 | 0.0333 |
| 133619 | PRRC1 | 1.40 | 0.0337 |
| 4121 | MAN1A1 | 1.60 | 0.0340 |
| **941** | **CD80** | **1.53** | **0.0350** |
| 2632 | GBE1 | 1.28 | 0.0353 |
| 6787 | NEK4 | 1.34 | 0.0354 |
| 64844 | MARCH7 | 1.27 | 0.0354 |
| 23347 | SMCHD1 | 1.29 | 0.0354 |
| 54431 | DNAJC10 | 1.25 | 0.0354 |
| 114885 | OSBPL11 | 1.32 | 0.0358 |
| **10342** | **TFG** | **1.28** | **0.0358** |
| 2553 | GABPB1 | 1.39 | 0.0358 |
| **6880** | **TAF9** | **1.29** | **0.0362** |
| **3838** | **KPNA2** | **1.52** | **0.0362** |
| **8673** | **VAMP8** | **1.58** | **0.0371** |
| 26031 | OSBPL3 | 1.43 | 0.0380 |
| **3162** | **HMOX1** | **2.26** | **0.0380** |
| 6120 | RPE | 1.21 | 0.0383 |
| **7099** | **TLR4** | **1.47** | **0.0383** |
| **10461** | **MERTK** | **1.43** | **0.0383** |
| 717 | C2 | 1.50 | 0.0383 |
| 29887 | SNX10 | 1.61 | 0.0383 |
| 54716 | SLC6A20 | 1.25 | 0.0383 |
| **1147** | **CHUK** | **1.63** | **0.0390** |
| 54969 | C4orf27 | 1.34 | 0.0399 |
| 55500 | ETNK1 | 1.44 | 0.0400 |
| 79157 | MFSD11 | 1.33 | 0.0400 |
| **912** | **CD1D** | **1.60** | **0.0400** |
| 10990 | LILRB5 | 1.29 | 0.0407 |
| **79071** | **ELOVL6** | **1.59** | **0.0407** |
| 55754 | TMEM30A | 1.37 | 0.0409 |
| **308** | **ANXA5** | **1.40** | **0.0409** |
| **5728** | **PTEN** | **1.27** | **0.0409** |
| 3597 | IL13RA1 | 1.38 | 0.0409 |
| **3459** | **IFNGR1** | **1.47** | **0.0409** |
| **51311** | **TLR8** | **1.56** | **0.0409** |
| **3689** | **ITGB2** | **1.90** | **0.0409** |
| 9874 | TLK1 | 1.24 | 0.0409 |
| 159 | ADSS | 1.30 | 0.0409 |
| 9055 | PRC1 | 2.61 | 0.0420 |
| 7462 | LAT2 | 1.56 | 0.0420 |
| 55247 | NEIL3 | 1.39 | 0.0420 |
| **3308** | **HSPA4** | **1.24** | **0.0420** |
| 8826 | IQGAP1 | 1.22 | 0.0420 |
| 23463 | ICMT | 1.23 | 0.0420 |
| 81930 | KIF18A | 1.34 | 0.0420 |
| **948** | **CD36** | **1.95** | **0.0420** |
| **3687** | **ITGAX** | **1.51** | **0.0426** |
| **7351** | **UCP2** | **1.69** | **0.0428** |
| **4973** | **OLR1** | **2.82** | **0.0428** |
| 9833 | MELK | 1.78 | 0.0428 |
| 10473 | HMGN4 | 1.34 | 0.0428 |
| 5660 | PSAP | 1.22 | 0.0428 |
| **9768** | **KIAA0101** | **1.50** | **0.0428** |
| 10288 | LILRB2 | 1.57 | 0.0428 |
| 4591 | TRIM37 | 1.28 | 0.0429 |
| 1654 | DDX3X | 1.25 | 0.0429 |
| 60343 | FAM3A | 0.65 | 0.0430 |
| 4542 | MYO1F | 1.72 | 0.0431 |
| 9824 | ARHGAP11A | 1.46 | 0.0431 |
| 22868 | FASTKD2 | 1.24 | 0.0431 |
| **51435** | **SCARA3** | **1.52** | **0.0431** |
| 4125 | MAN2B1 | 1.48 | 0.0431 |
| 1880 | GPR183 | 2.59 | 0.0431 |
| 25978 | CHMP2B | 1.26 | 0.0431 |
| 9837 | GINS1 | 2.28 | 0.0432 |
| 79647 | AKIRIN1 | 1.37 | 0.0432 |
| 8445 | DYRK2 | 1.36 | 0.0434 |
| 55740 | ENAH | 1.44 | 0.0435 |
| 9562 | MINPP1 | 1.47 | 0.0435 |
| 3796 | KIF2A | 1.37 | 0.0463 |
| 10659 | CUGBP2 | 1.44 | 0.0463 |
| **9435** | **CHST2** | **1.89** | **0.0463** |
| **5744** | **PTHLH** | **1.72** | **0.0463** |
| 55839 | CENPN | 1.26 | 0.0463 |
| **1832** | **DSP** | **2.37** | **0.0463** |
| 6036 | RNASE2 | 2.43 | 0.0463 |
| 25994 | HIGD1A | 1.36 | 0.0463 |
| **9516** | **LITAF** | **1.30** | **0.0463** |
| **4893** | **NRAS** | **1.53** | **0.0463** |
| **114548** | **NLRP3** | **1.20** | **0.0463** |
| 951 | CD37 | 1.59 | 0.0463 |
| 7690 | ZNF131 | 1.31 | 0.0463 |
| 10015 | PDCD6IP | 1.28 | 0.0463 |
| 55916 | NXT2 | 1.28 | 0.0463 |
| 51379 | CRLF3 | 1.35 | 0.0463 |
| **5341** | **PLEK** | **1.66** | **0.0463** |
| 51313 | C4orf18 | 1.41 | 0.0463 |
| **7097** | **TLR2** | **1.79** | **0.0463** |
| **7035** | **TFPI** | **1.36** | **0.0465** |
| 8099 | CDK2AP1 | 1.16 | 0.0465 |
| 65084 | TMEM135 | 1.50 | 0.0465 |
| **140** | **ADORA3** | **1.69** | **0.0465** |
| 57103 | C12orf5 | 1.48 | 0.0465 |
| **6303** | **SAT1** | **1.35** | **0.0465** |
| **51290** | **ERGIC2** | **1.31** | **0.0465** |
| 4076 | CAPRIN1 | 1.24 | 0.0465 |
| 865 | CBFB | 1.36 | 0.0465 |
| 81671 | TMEM49 | 1.36 | 0.0465 |
| **4318** | **MMP9** | **2.68** | **0.0465** |
| 10159 | ATP6AP2 | 1.33 | 0.0465 |
| 30001 | ERO1L | 1.29 | 0.0479 |
| 5791 | PTPRE | 1.69 | 0.0479 |
| 11196 | SEC23IP | 1.13 | 0.0487 |
| **241** | **ALOX5AP** | **2.04** | **0.0487** |
| 10550 | ARL6IP5 | 1.25 | 0.0487 |
| 5250 | SLC25A3 | 1.16 | 0.0489 |
| 27299 | ADAMDEC1 | 1.83 | 0.0489 |
| 55215 | FANCI | 1.40 | 0.0491 |
| 5552 | SRGN | 1.58 | 0.0491 |
| **1356** | **CP** | **1.52** | **0.0491** |
| **11221** | **DUSP10** | **1.30** | **0.0491** |
