# Supplementary material for: The Molecular Phenotype of Endocapillary Proliferation: Novel Therapeutic Targets for IgA Nephropathy
Source: PLoS One. 2014 Aug 18;9(8):e103413. doi: 10.1371/journal.pone.0103413 (PMC4136785; doi:10.1371/journal.pone.0103413)
Supplement: Table S3 — Considering endocapillary proliferation as a continuous variable. As illustrated in Figure S1, we also correlated mRNA expression with endocapillary proliferation severity (ie. endocapillary proliferation considered as a continuous variable). With this approach we identified 141 transcripts that are: a – differentially expressed in E1 vs. E0 biopsies and b – correlated with the the relative degree of endocapillary proliferation. (DOCX) [file pone.0103413.s004.docx]

**Supplementary Table S3.** Considering endocapillary proliferation as a continuous variable.

As illustrated in Supplementary Figure S1, we also correlated mRNA expression with endocapillary proliferation severity (ie. endocapillary proliferation considered as a continuous variable). With this approach we identified 141 transcripts that are: a – differentially expressed in E1 vs. E0 biopsies and b – correlated with the the relative degree of endocapillary proliferation. This list is provided below.

| GeneID | Symbol | Full name | p value | FDR | r | r2 |
| --- | --- | --- | --- | --- | --- | --- |
| 19 | ABCA1 | ATP-binding cassette, sub-family A (ABC1), member 1 | 0.000 | 0.010 | 0.74 | 0.55 |
| 140 | ADORA3 | adenosine A3 receptor | 0.000 | 0.000 | 0.78 | 0.61 |
| 332 | BIRC5 | baculoviral IAP repeat containing 5 | 0.000 | 0.040 | 0.67 | 0.45 |
| 341 | APOC1 | apolipoprotein C-I | 0.000 | 0.030 | 0.70 | 0.49 |
| 387 | RHOA | ras homolog family member A | 0.000 | 0.040 | 0.66 | 0.43 |
| 526 | ATP6V1B2 | ATPase, H+ transporting, lysosomal 56/58kDa, V1 subunit B2 | 0.000 | 0.040 | 0.67 | 0.44 |
| 586 | BCAT1 | branched chain amino-acid transaminase 1, cytosolic | 0.000 | 0.000 | 0.79 | 0.62 |
| 712 | C1QA | complement component 1, q subcomponent, A chain | 0.000 | 0.000 | 0.86 | 0.74 |
| 713 | C1QB | complement component 1, q subcomponent, B chain | 0.000 | 0.000 | 0.82 | 0.67 |
| 717 | C2 | complement component 2 | 0.000 | 0.010 | 0.73 | 0.54 |
| 728 | C5AR1 | complement component 5a receptor 1 | 0.000 | 0.000 | 0.78 | 0.61 |
| 920 | CD4 | CD4 molecule | 0.000 | 0.030 | 0.69 | 0.48 |
| 929 | CD14 | CD14 molecule | 0.000 | 0.010 | 0.76 | 0.58 |
| 960 | CD44 | CD44 molecule (Indian blood group) | 0.000 | 0.030 | 0.69 | 0.48 |
| 983 | CDK1 | cyclin-dependent kinase 1 | 0.000 | 0.010 | 0.77 | 0.59 |
| 1033 | CDKN3 | cyclin-dependent kinase inhibitor 3 | 0.000 | 0.030 | 0.70 | 0.49 |
| 1058 | CENPA | centromere protein A | 0.000 | 0.040 | 0.67 | 0.45 |
| 1063 | CENPF | centromere protein F, 350/400kDa | 0.000 | 0.020 | 0.70 | 0.49 |
| 1230 | CCR1 | chemokine (C-C motif) receptor 1 | 0.000 | 0.000 | 0.79 | 0.62 |
| 1436 | CSF1R | colony stimulating factor 1 receptor | 0.000 | 0.010 | 0.73 | 0.54 |
| 1520 | CTSS | cathepsin S | 0.000 | 0.020 | 0.71 | 0.50 |
| 1536 | CYBB | cytochrome b-245, beta polypeptide | 0.000 | 0.020 | 0.72 | 0.51 |
| 1794 | DOCK2 | dedicator of cytokinesis 2 | 0.000 | 0.010 | 0.78 | 0.61 |
| 1880 | GPR183 | G protein-coupled receptor 183 | 0.000 | 0.020 | 0.72 | 0.52 |
| 2123 | EVI2A | ecotropic viral integration site 2A | 0.000 | 0.010 | 0.73 | 0.54 |
| 2124 | EVI2B | ecotropic viral integration site 2B | 0.000 | 0.020 | 0.70 | 0.49 |
| 2146 | EZH2 | enhancer of zeste homolog 2 (Drosophila) | 0.000 | 0.040 | 0.66 | 0.44 |
| 2161 | F12 | coagulation factor XII (Hageman factor) | 0.000 | 0.000 | 0.80 | 0.64 |
| 2180 | ACSL1 | acyl-CoA synthetase long-chain family member 1 | 0.000 | 0.030 | 0.69 | 0.47 |
| 2359 | FPR3 | formyl peptide receptor 3 | 0.000 | 0.000 | 0.90 | 0.81 |
| 2585 | GALK2 | galactokinase 2 | 0.000 | 0.030 | 0.68 | 0.47 |
| 2799 | GNS | glucosamine (N-acetyl)-6-sulfatase | 0.000 | 0.040 | 0.65 | 0.43 |
| 3015 | H2AFZ | H2A histone family, member Z | 0.000 | 0.030 | 0.69 | 0.47 |
| 3059 | HCLS1 | hematopoietic cell-specific Lyn substrate 1 | 0.000 | 0.040 | 0.65 | 0.43 |
| 3071 | NCKAP1L | NCK-associated protein 1-like | 0.000 | 0.000 | 0.79 | 0.62 |
| 3074 | HEXB | hexosaminidase B (beta polypeptide) | 0.000 | 0.010 | 0.75 | 0.56 |
| 3161 | HMMR | hyaluronan-mediated motility receptor (RHAMM) | 0.000 | 0.030 | 0.70 | 0.48 |
| 3162 | HMOX1 | heme oxygenase (decycling) 1 | 0.000 | 0.010 | 0.77 | 0.59 |
| 3308 | HSPA4 | heat shock 70kDa protein 4 | 0.000 | 0.030 | 0.70 | 0.49 |
| 3587 | IL10RA | interleukin 10 receptor, alpha | 0.000 | 0.030 | 0.69 | 0.48 |
| 3590 | IL11RA | interleukin 11 receptor, alpha | 0.000 | 0.020 | -0.72 | 0.52 |
| 3597 | IL13RA1 | interleukin 13 receptor, alpha 1 | 0.000 | 0.030 | 0.70 | 0.48 |
| 3676 | ITGA4 | integrin, alpha 4 (antigen CD49D, alpha 4 subunit of VLA-4 receptor) | 0.000 | 0.000 | 0.83 | 0.69 |
| 3684 | ITGAM | integrin, alpha M (complement component 3 receptor 3 subunit) | 0.000 | 0.020 | 0.71 | 0.50 |
| 3687 | ITGAX | integrin, alpha X (complement component 3 receptor 4 subunit) | 0.000 | 0.030 | 0.68 | 0.46 |
| 3832 | KIF11 | kinesin family member 11 | 0.000 | 0.040 | 0.67 | 0.44 |
| 3903 | LAIR1 | leukocyte-associated immunoglobulin-like receptor 1 | 0.000 | 0.010 | 0.78 | 0.60 |
| 3936 | LCP1 | lymphocyte cytosolic protein 1 (L-plastin) | 0.000 | 0.030 | 0.69 | 0.48 |
| 3988 | LIPA | lipase A, lysosomal acid, cholesterol esterase | 0.000 | 0.010 | 0.77 | 0.60 |
| 4082 | MARCKS | myristoylated alanine-rich protein kinase C substrate | 0.000 | 0.030 | 0.68 | 0.46 |
| 4121 | MAN1A1 | mannosidase, alpha, class 1A, member 1 | 0.000 | 0.030 | 0.69 | 0.48 |
| 4144 | MAT2A | methionine adenosyltransferase II, alpha | 0.000 | 0.000 | 0.80 | 0.63 |
| 4200 | ME2 | malic enzyme 2, NAD(+)-dependent, mitochondrial | 0.000 | 0.030 | 0.69 | 0.47 |
| 4288 | MKI67 | marker of proliferation Ki-67 | 0.000 | 0.020 | 0.72 | 0.52 |
| 4318 | MMP9 | matrix metallopeptidase 9 (gelatinase B, 92kDa gelatinase, 92kDa type IV collagenase) | 0.000 | 0.010 | 0.76 | 0.58 |
| 4321 | MMP12 | matrix metallopeptidase 12 (macrophage elastase) | 0.000 | 0.000 | 0.81 | 0.65 |
| 4481 | MSR1 | macrophage scavenger receptor 1 | 0.000 | 0.000 | 0.91 | 0.82 |
| 4811 | NID1 | nidogen 1 | 0.000 | 0.000 | 0.83 | 0.69 |
| 5341 | PLEK | pleckstrin | 0.000 | 0.030 | 0.70 | 0.49 |
| 5594 | MAPK1 | mitogen-activated protein kinase 1 | 0.000 | 0.030 | 0.68 | 0.46 |
| 5728 | PTEN | phosphatase and tensin homolog | 0.000 | 0.030 | 0.68 | 0.46 |
| 5791 | PTPRE | protein tyrosine phosphatase, receptor type, E | 0.000 | 0.030 | 0.69 | 0.48 |
| 5873 | RAB27A | RAB27A, member RAS oncogene family | 0.000 | 0.030 | 0.69 | 0.48 |
| 5888 | RAD51 | RAD51 recombinase | 0.000 | 0.010 | 0.75 | 0.57 |
| 6036 | RNASE2 | ribonuclease, RNase A family, 2 (liver, eosinophil-derived neurotoxin) | 0.000 | 0.020 | 0.71 | 0.50 |
| 6039 | RNASE6 | ribonuclease, RNase A family, k6 | 0.000 | 0.000 | 0.83 | 0.69 |
| 6059 | ABCE1 | ATP-binding cassette, sub-family E (OABP), member 1 | 0.000 | 0.030 | 0.69 | 0.48 |
| 6241 | RRM2 | ribonucleotide reductase M2 | 0.000 | 0.010 | 0.74 | 0.55 |
| 6856 | SYPL1 | synaptophysin-like 1 | 0.000 | 0.040 | 0.66 | 0.44 |
| 7035 | TFPI | tissue factor pathway inhibitor (lipoprotein-associated coagulation inhibitor) | 0.000 | 0.040 | 0.66 | 0.43 |
| 7045 | TGFBI | transforming growth factor, beta-induced, 68kDa | 0.000 | 0.040 | 0.66 | 0.44 |
| 7096 | TLR1 | toll-like receptor 1 | 0.000 | 0.010 | 0.76 | 0.58 |
| 7153 | TOP2A | topoisomerase (DNA) II alpha 170kDa | 0.000 | 0.030 | 0.70 | 0.48 |
| 7305 | TYROBP | TYRO protein tyrosine kinase binding protein | 0.000 | 0.040 | 0.67 | 0.44 |
| 7351 | UCP2 | uncoupling protein 2 (mitochondrial, proton carrier) | 0.000 | 0.030 | 0.70 | 0.49 |
| 7456 | WIPF1 | WAS/WASL interacting protein family, member 1 | 0.000 | 0.000 | 0.79 | 0.62 |
| 7803 | PTP4A1 | protein tyrosine phosphatase type IVA, member 1 | 0.000 | 0.000 | 0.82 | 0.67 |
| 7805 | LAPTM5 | lysosomal protein transmembrane 5 | 0.000 | 0.020 | 0.71 | 0.51 |
| 7941 | PLA2G7 | phospholipase A2, group VII (platelet-activating factor acetylhydrolase, plasma) | 0.000 | 0.010 | 0.77 | 0.59 |
| 8477 | GPR65 | G protein-coupled receptor 65 | 0.000 | 0.020 | 0.72 | 0.51 |
| 8522 | GAS7 | growth arrest-specific 7 | 0.000 | 0.040 | 0.66 | 0.43 |
| 8869 | ST3GAL5 | ST3 beta-galactoside alpha-2,3-sialyltransferase 5 | 0.000 | 0.000 | 0.81 | 0.66 |
| 8914 | TIMELESS | timeless circadian clock | 0.000 | 0.040 | 0.66 | 0.43 |
| 8942 | KYNU | kynureninase | 0.000 | 0.020 | 0.72 | 0.52 |
| 9050 | PSTPIP2 | proline-serine-threonine phosphatase interacting protein 2 | 0.000 | 0.040 | 0.67 | 0.45 |
| 9180 | OSMR | oncostatin M receptor | 0.000 | 0.040 | 0.67 | 0.45 |
| 9232 | PTTG1 | pituitary tumor-transforming 1 | 0.000 | 0.020 | 0.70 | 0.50 |
| 9332 | CD163 | CD163 molecule | 0.000 | 0.000 | 0.84 | 0.70 |
| 9493 | KIF23 | kinesin family member 23 | 0.000 | 0.040 | 0.67 | 0.45 |
| 9510 | ADAMTS1 | ADAM metallopeptidase with thrombospondin type 1 motif, 1 | 0.000 | 0.040 | 0.65 | 0.43 |
| 9555 | H2AFY | H2A histone family, member Y | 0.000 | 0.000 | 0.80 | 0.64 |
| 9770 | RASSF2 | Ras association (RalGDS/AF-6) domain family member 2 | 0.000 | 0.010 | 0.73 | 0.54 |
| 10112 | KIF20A | kinesin family member 20A | 0.000 | 0.030 | 0.68 | 0.47 |
| 10184 | LHFPL2 | lipoma HMGIC fusion partner-like 2 | 0.000 | 0.020 | 0.73 | 0.53 |
| 10228 | STX6 | syntaxin 6 | 0.000 | 0.010 | 0.77 | 0.59 |
| 10434 | LYPLA1 | lysophospholipase I | 0.000 | 0.020 | 0.72 | 0.52 |
| 10461 | MERTK | c-mer proto-oncogene tyrosine kinase | 0.000 | 0.000 | 0.82 | 0.66 |
| 10527 | IPO7 | importin 7 | 0.000 | 0.040 | 0.67 | 0.44 |
| 10855 | HPSE | heparanase | 0.000 | 0.000 | 0.79 | 0.62 |
| 10981 | RAB32 | RAB32, member RAS oncogene family | 0.000 | 0.010 | 0.76 | 0.58 |
| 10990 | LILRB5 | leukocyte immunoglobulin-like receptor, subfamily B (with TM and ITIM domains), member 5 | 0.000 | 0.020 | 0.72 | 0.51 |
| 11326 | VSIG4 | V-set and immunoglobulin domain containing 4 | 0.000 | 0.000 | 0.85 | 0.72 |
| 23484 | LEPROTL1 | leptin receptor overlapping transcript-like 1 | 0.000 | 0.040 | 0.66 | 0.44 |
| 23603 | CORO1C | coronin, actin binding protein, 1C | 0.000 | 0.020 | 0.72 | 0.51 |
| 25939 | SAMHD1 | SAM domain and HD domain 1 | 0.000 | 0.040 | 0.67 | 0.45 |
| 26191 | PTPN22 | protein tyrosine phosphatase, non-receptor type 22 (lymphoid) | 0.000 | 0.000 | 0.80 | 0.64 |
| 27299 | ADAMDEC1 | ADAM-like, decysin 1 | 0.000 | 0.010 | 0.76 | 0.57 |
| 29015 | SLC43A3 | solute carrier family 43, member 3 | 0.000 | 0.000 | 0.84 | 0.70 |
| 29028 | ATAD2 | ATPase family, AAA domain containing 2 | 0.000 | 0.030 | 0.69 | 0.48 |
| 29887 | SNX10 | sorting nexin 10 | 0.000 | 0.040 | 0.65 | 0.43 |
| 51167 | CYB5R4 | cytochrome b5 reductase 4 | 0.000 | 0.020 | 0.72 | 0.52 |
| 51203 | NUSAP1 | nucleolar and spindle associated protein 1 | 0.000 | 0.040 | 0.67 | 0.45 |
| 51311 | TLR8 | toll-like receptor 8 | 0.000 | 0.010 | 0.75 | 0.57 |
| 51313 | FAM198B | family with sequence similarity 198, member B | 0.000 | 0.010 | 0.75 | 0.57 |
| 51338 | MS4A4A | membrane-spanning 4-domains, subfamily A, member 4A | 0.000 | 0.000 | 0.88 | 0.78 |
| 51514 | DTL | denticleless E3 ubiquitin protein ligase homolog (Drosophila) | 0.000 | 0.030 | 0.68 | 0.46 |
| 51571 | FAM49B | family with sequence similarity 49, member B | 0.000 | 0.030 | 0.69 | 0.47 |
| 51714 | SELT | - | 0.000 | 0.020 | 0.71 | 0.50 |
| 51762 | RAB8B | RAB8B, member RAS oncogene family | 0.000 | 0.000 | 0.81 | 0.66 |
| 54149 | C21orf91 | chromosome 21 open reading frame 91 | 0.000 | 0.030 | 0.69 | 0.48 |
| 54478 | FAM64A | family with sequence similarity 64, member A | 0.000 | 0.030 | 0.69 | 0.47 |
| 54491 | FAM105A | family with sequence similarity 105, member A | 0.000 | 0.000 | 0.82 | 0.68 |
| 54716 | SLC6A20 | solute carrier family 6 (proline IMINO transporter), member 20 | 0.000 | 0.030 | 0.69 | 0.47 |
| 55355 | HJURP | Holliday junction recognition protein | 0.000 | 0.010 | 0.78 | 0.60 |
| 55379 | LRRC59 | leucine rich repeat containing 59 | 0.000 | 0.040 | 0.66 | 0.44 |
| 55872 | PBK | PDZ binding kinase | 0.000 | 0.030 | 0.68 | 0.47 |
| 56992 | KIF15 | kinesin family member 15 | 0.000 | 0.020 | 0.71 | 0.51 |
| 57405 | SPC25 | SPC25, NDC80 kinetochore complex component | 0.000 | 0.010 | 0.75 | 0.57 |
| 58504 | ARHGAP22 | Rho GTPase activating protein 22 | 0.000 | 0.030 | 0.69 | 0.48 |
| 64092 | SAMSN1 | SAM domain, SH3 domain and nuclear localization signals 1 | 0.000 | 0.030 | 0.68 | 0.47 |
| 64116 | SLC39A8 | solute carrier family 39 (zinc transporter), member 8 | 0.000 | 0.000 | 0.84 | 0.71 |
| 64151 | NCAPG | non-SMC condensin I complex, subunit G | 0.000 | 0.030 | 0.68 | 0.46 |
| 64231 | MS4A6A | membrane-spanning 4-domains, subfamily A, member 6A | 0.000 | 0.010 | 0.77 | 0.59 |
| 64866 | CDCP1 | CUB domain containing protein 1 | 0.000 | 0.030 | 0.68 | 0.47 |
| 79666 | PLEKHF2 | pleckstrin homology domain containing, family F (with FYVE domain) member 2 | 0.000 | 0.040 | 0.67 | 0.45 |
| 79709 | COLGALT1 | collagen beta(1-O)galactosyltransferase 1 | 0.000 | 0.010 | 0.77 | 0.59 |
| 80183 | KIAA0226L | KIAA0226-like | 0.000 | 0.030 | 0.69 | 0.47 |
| 80896 | NPL | N-acetylneuraminate pyruvate lyase (dihydrodipicolinate synthase) | 0.000 | 0.020 | 0.72 | 0.52 |
| 81553 | FAM49A | family with sequence similarity 49, member A | 0.000 | 0.000 | 0.88 | 0.78 |
| 81671 | VMP1 | vacuole membrane protein 1 | 0.000 | 0.040 | 0.66 | 0.44 |
| 259266 | ASPM | asp (abnormal spindle) homolog, microcephaly associated (Drosophila) | 0.000 | 0.040 | 0.66 | 0.44 |
