# Supplementary material for: The Molecular Phenotype of Endocapillary Proliferation: Novel Therapeutic Targets for IgA Nephropathy
Source: PLoS One. 2014 Aug 18;9(8):e103413. doi: 10.1371/journal.pone.0103413 (PMC4136785; doi:10.1371/journal.pone.0103413)
Supplement: Table S4 — Canonical pathways significantly regulated (p-value<0.05) from the 424 genes regulated in E1 vs. E0 biopsies, as assessed by IPA (Ingenuity Pathway Analysis). (DOCX) [file pone.0103413.s005.docx]

**Supplementary Table S4.** Canonical pathways significantly regulated (p-value<0.05) from the 424 genes regulated in E1 vs. E0 biopsies, as assessed by IPA (Ingenuity Pathway Analysis).

| **Canonical pathways (number of genes regulated/number of genes in the pathway)** | **p-value** | **Regulated molecules in the pathway** |
| --- | --- | --- |
| **Role of Pattern Recognition Receptors in Recognition of Bacteria and Viruses (14/106)** | 0.0000 | PTX3,TLR1,PIK3CA,NLRP3,MAPK1,TLR8,C1QA,C1QB,EIF2S1,TLR2,TLR4,NOD2,C5AR1,PRKD3 |
| **Leukocyte Extravasation Signaling (18/201)** | 0.0001 | PIK3CA,MAPK1,ITGA2,BTK,ITGB2,WIPF1,ITGAM,TIMP1,RHOA,NCF2,CD44,CYBB,MMP12,PRKD3,MMP9,MMP1,ITGA4,ITK |
| **TREM1 Signaling (9/71)** | 0.0001 | TLR2,TLR4,TLR1,NOD2,MAPK1,TYROBP,TLR8,LAT2,ITGAX |
| **NF-κB Activation by Viruses (10/82)** | 0.0003 | ITGB2,PIK3CA,CCR5,NRAS,MAPK1,CD4,ITGA2,CHUK,PRKD3,ITGA4 |
| **Toll-like Receptor Signaling (8/62)** | 0.0004 | TLR2,TLR4,TLR1,MAPK1,MAP3K7,TLR8,CD14,CHUK |
| **Cell Cycle: G2/M DNA Damage Checkpoint Regulation (7/48)** | 0.0006 | YWHAH,YWHAZ,TOP2A,CCNB2,CDK1,CHEK1,CCNB1 |
| **IL-8 Signaling (16/205)** | 0.0006 | PIK3CA,NRAS,MAPK1,IQGAP1,RAB11FIP2,ITGB2,HMOX1,ITGAM,RHOA,NCF2,CYBB,CHUK,PRKD3,GNG12,MMP9,ITGAX |
| **Glioma Invasiveness Signaling (8/60)** | 0.0007 | PIK3CA,NRAS,MAPK1,TIMP1,HMMR,RHOA,CD44,MMP9 |
| **Mitotic Roles of Polo-Like Kinase (8/70)** | 0.0008 | KIF23,CDC20,PTTG1,PRC1,CCNB2,CDK1,KIF11,CCNB1 |
| **Rac Signaling (11/122)** | 0.0009 | ACTR2,PIK3CA,NRAS,MAPK1,RHOA,NCF2,ITGA2,CD44,CYBB,IQGAP1,ITGA4 |
| **MSP-RON Signaling Pathway (7/50)** | 0.0010 | TLR2,ITGB2,TLR4,F12,PIK3CA,ITGAM,CCR2 |
| **T Cell Receptor Signaling (10/109)** | 0.0016 | PTPRC,BTK,CD3G,PIK3CA,NRAS,MAPK1,CD4,CHUK,RASA1,ITK |
| **Inhibition of Matrix Metalloproteases (6/40)** | 0.0019 | ADAM12,TIMP1,ADAM10,MMP12,MMP9,MMP1 |
| **IL-10 Signaling (8/78)** | 0.0029 | CCR1,HMOX1,CCR5,MAPK1,MAP3K7,IL10RA,CD14,CHUK |
| **Macropinocytosis Signaling (8/76)** | 0.0029 | ITGB2,PIK3CA,NRAS,ABI1,RHOA,CD14,PRKD3,CSF1R |
| **LPS-stimulated MAPK Signaling (8/82)** | 0.0049 | TLR4,PIK3CA,NRAS,MAPK1,MAP3K7,CD14,CHUK,PRKD3 |
| **Hereditary Breast Cancer Signaling (10/128)** | 0.0054 | RAD51,RB1,PIK3CA,NRAS,MSH6,HLTF,CDK1,PTEN,CHEK1,CCNB1 |
| **Myo-inositol Biosynthesis (2/8)** | 0.0078 | IMPA1,IMPAD1 |
| **Production of Nitric Oxide and Reactive Oxygen Species in Macrophages (13/210)** | 0.0089 | PIK3CA,MAPK1,IFNGR1,TLR2,APOC1,TLR4,MAP3K7,RHOA,NCF2,CYBB,IRF8,CHUK,PRKD3 |
| **Superpathway of D-myo-inositol (1,4,5)-trisphosphate Metabolism (4/33)** | 0.0093 | MINPP1,IMPA1,IMPAD1,PTEN |
| **Neuregulin Signaling (8/102)** | 0.0100 | NRAS,MAPK1,ITGA2,PRKD3,EREG,PTEN,ITGA4,PSEN1 |
| **Actin Nucleation by ARP-WASP Complex (6/66)** | 0.0120 | ACTR2,WIPF1,NRAS,RHOA,ITGA2,ITGA4 |
| **Regulation of Cellular Mechanics by Calpain Protease (6/72)** | 0.0120 | RB1,NRAS,MAPK1,ITGA2,CDK1,ITGA4 |
| **fMLP Signaling in Neutrophils (9/129)** | 0.0123 | FPR3,ACTR2,PIK3CA,NRAS,MAPK1,NCF2,CYBB,PRKD3,GNG12 |
| **Lipid Antigen Presentation by CD1 (3/23)** | 0.0135 | PSAP,CANX,CD1D |
| **Colorectal Cancer Metastasis Signaling (15/258)** | 0.0138 | TLR1,PIK3CA,NRAS,MAPK1,TLR8,IFNGR1,BIRC5,TLR2,TLR4,RHOA,MSH6,MMP12,MMP1,GNG12,MMP9 |
| **ATM Signaling (6/61)** | 0.0141 | RAD51,CCNB2,TLK1,CDK1,CHEK1,CCNB1 |
| **Melanoma Signaling (5/46)** | 0.0170 | RB1,PIK3CA,NRAS,MAPK1,PTEN |
| **iNOS Signaling (5/53)** | 0.0170 | TLR4,MAPK1,CD14,IFNGR1,CHUK |
| **Extrinsic Prothrombin Activation Pathway (3/20)** | 0.0195 | F12,PROS1,TFPI |
| **Atherosclerosis Signaling (9/136)** | 0.0204 | APOC1,ITGB2,MSR1,CD36,CCR2,PLA2G7,MMP9,MMP1,ITGA4 |
| **iCOS-iCOSL Signaling in T Helper Cells (8/123)** | 0.0214 | PTPRC,CD3G,PIK3CA,CD80,CD4,CHUK,PTEN,ITK |
| **Paxillin Signaling (8/111)** | 0.0229 | ITGB2,PIK3CA,ITGAM,NRAS,MAPK1,ITGA2,ITGA4,ITGAX |
| **CCR5 Signaling in Macrophages (6/95)** | 0.0245 | CD3G,CCR5,MAPK1,CD4,PRKD3,GNG12 |
| **Cdc42 Signaling (9/177)** | 0.0251 | CD3G,ACTR2,WIPF1,MAPK1,ITGA2,IQGAP1,RASA1,ITGA4,ITK |
| **PI3K/AKT Signaling (9/144)** | 0.0251 | PIK3CA,NRAS,MAPK1,YWHAH,ITGA2,YWHAZ,CHUK,PTEN,ITGA4 |
| **Complement System (4/35)** | 0.0263 | C5AR1,C1QA,C1QB,C2 |
| **DNA damage-induced 14-3-3σ Signaling (3/21)** | 0.0269 | CCNB2,CDK1,CCNB1 |
| **Polyamine Regulation in Colon Cancer (3/29)** | 0.0316 | AZIN1,SAT1,PSME3 |
| **GADD45 Signaling (3/22)** | 0.0316 | PCNA,CDK1,CCNB1 |
| **IL-17A Signaling in Fibroblasts (4/40)** | 0.0324 | MAPK1,MAP3K7,CHUK,MMP1 |
| **Agrin Interactions at Neuromuscular Junction (6/69)** | 0.0324 | ITGB2,GABPB1,NRAS,MAPK1,ITGA2,ITGA4 |
| **Airway Pathology in Chronic Obstructive Pulmonary Disease (2/9)** | 0.0331 | MMP9,MMP1 |
| **FAK Signaling (7/101)** | 0.0339 | PIK3CA,NRAS,MAPK1,HMMR,ITGA2,PTEN,ITGA4 |
| **Communication between Innate and Adaptive Immune Cells (6/109)** | 0.0347 | TLR2,TLR4,TLR1,CD80,CD4,TLR8 |
| **PKCθ Signaling in T Lymphocytes (8/143)** | 0.0347 | CD3G,PIK3CA,NRAS,CD80,MAPK1,MAP3K7,CD4,CHUK |
| **Ephrin Receptor Signaling (11/201)** | 0.0347 | ACTR2,WIPF1,NRAS,ABI1,MAPK1,RHOA,ITGA2,ADAM10,RASA1,GNG12,ITGA4 |
| **Oncostatin M Signaling (4/35)** | 0.0355 | NRAS,MAPK1,OSMR,MMP1 |
| **Ovarian Cancer Signaling (9/142)** | 0.0372 | RAD51,RB1,PIK3CA,NRAS,MAPK1,MSH6,CD44,MMP9,PTEN |
| **CD28 Signaling in T Helper Cells (8/132)** | 0.0389 | PTPRC,CD3G,ACTR2,PIK3CA,CD80,CD4,CHUK,ITK |
| **Coagulation System (4/38)** | 0.0389 | F12,PROS1,TFPI,SERPINE1 |
| **Integrin Signaling (12/207)** | 0.0407 | ITGB2,ACTR2,PIK3CA,WIPF1,ITGAM,NRAS,MAPK1,RHOA,ITGA2,PTEN,ITGA4,ITGAX |
| **Pathogenesis of Multiple Sclerosis (2/9)** | 0.0417 | CCR1,CCR5 |
| **Fcγ Receptor-mediated Phagocytosis in Macrophages and Monocytes (7/102)** | 0.0417 | MYO5A,HMOX1,ACTR2,YES1,MAPK1,PRKD3,PTEN |
| **Mechanisms of Viral Exit from Host Cells (4/45)** | 0.0427 | CHMP2B,PDCD6IP,PRKD3,LMNB1 |
| **p53 Signaling (7/96)** | 0.0437 | RB1,PCNA,PIK3CA,C12orf5,PTEN,BIRC5,CHEK1 |
| **Altered T Cell and B Cell Signaling in Rheumatoid Arthritis (6/92)** | 0.0447 | TLR2,TLR4,TLR1,CD80,TLR8,CHUK |
| **PEDF Signaling (6/78)** | 0.0447 | ARHGAP22,PIK3CA,NRAS,MAPK1,RHOA,CHUK |
| **HMGB1 Signaling (7/99)** | 0.0468 | TLR4,PIK3CA,NRAS,MAPK1,RHOA,IFNGR1,SERPINE1 |
| **IL-12 Signaling and Production in Macrophages (9/156)** | 0.0479 | TLR2,APOC1,TLR4,PIK3CA,MAPK1,IFNGR1,IRF8,CHUK,PRKD3 |
