# Supplementary material for: The Molecular Phenotype of Endocapillary Proliferation: Novel Therapeutic Targets for IgA Nephropathy
Source: PLoS One. 2014 Aug 18;9(8):e103413. doi: 10.1371/journal.pone.0103413 (PMC4136785; doi:10.1371/journal.pone.0103413)
Supplement: Table S5 — 144 genes of the 424 regulated in E1 vs E0 have a binding site for NFKB1 in their promoter. (DOCX) [file pone.0103413.s006.docx]

**Supplementary Table S5.** 144 genes of the 424 regulated in E1 vs E0 have a binding site for NFKB1 in their promoter.

| ABCA1 | CD80 | IL10RA | NLRP3 | SLC38A2 |
| --- | --- | --- | --- | --- |
| ACSL1 | CDK1 | IRF8 | NOD2 | SLC39A8 |
| ACTR2 | CDKN3 | ITCH | NRAS | SRSF1 |
| ACVR1 | CHEK1 | ITGA2 | OLR1 | ST3GAL5 |
| ADAM10 | CHST2 | ITGA4 | PBK | TAF9 |
| ADAM12 | CHUK | ITGAM | PCNA | TBL1XR1 |
| ADAM9 | CP | ITGAX | PGGT1B | TFEC |
| ADAMTS1 | CSF1R | ITGB2 | PIK3CA | TFG |
| ADORA3 | CYBB | ITK | PLEK | TFPI |
| AIM2 | DEGS1 | KIAA0101 | PRDX3 | TIMP1 |
| ALOX5AP | DLGAP5 | KIF4A | PRNP | TLR1 |
| ANXA5 | DOCK2 | KPNA2 | PROS1 | TLR2 |
| APOC1 | DSC2 | LAIR1 | PSEN1 | TLR4 |
| ASCC3 | DSP | LAPTM5 | PTEN | TLR8 |
| ATAD2 | DUSP10 | LIF | PTHLH | TMPO |
| BIRC5 | EIF2S1 | LITAF | PTPRC | TNFAIP8 |
| BTK | ELOVL6 | LMNB1 | PTTG1 | TNFRSF21 |
| C1QA | EREG | MAP3K7 | PTX3 | TYMS |
| C5AR1 | ERGIC2 | MAPK1 | RAD51 | TYROBP |
| CANX | EZH2 | MAT2A | RASA1 | UCP2 |
| CCNB1 | FOSL1 | MERTK | RASSF2 | VAMP8 |
| CCNC | FOXM1 | MMD | RB1 | YES1 |
| CCNL1 | FUT4 | MMP1 | RHOA | YWHAH |
| CCR5 | G3BP1 | MMP9 | RRM2 | YWHAZ |
| CD163 | GLRX3 | MSH6 | SAMSN1 |  |
| CD1D | HMOX1 | MSR1 | SAT1 |  |
| CD36 | HPSE | MYO5A | SCARA3 |  |
| CD3G | HSPA4 | NAMPT | SERPINE1 |  |
| CD4 | IFNGR1 | NCF2 | SLC1A3 |  |
| CD44 | IGFBP1 | NCKAP1L | SLC30A5 |  |
