# Supplementary material for: The Molecular Phenotype of Endocapillary Proliferation: Novel Therapeutic Targets for IgA Nephropathy
Source: PLoS One. 2014 Aug 18;9(8):e103413. doi: 10.1371/journal.pone.0103413 (PMC4136785; doi:10.1371/journal.pone.0103413)
Supplement: Table S6 — Transcription factor analysis results. This list includes TFs able to bind to the promoter region of at least 30 different genes from the 424 genes differentially regulated in E1 vs E0. (DOCX) [file pone.0103413.s007.docx]

**Supplementary Table S6.** Transcription factor analysis results. This list includes TFs able to bind to the promoter region of at least 30 different genes from the 424 genes differentially regulated in E1 vs E0.

| **From 1495 Human Transcription factor list** | **# of genes in the 424 gene list having a binding site for the written TF** | **TF mRNA is differentially expressed E1 v E0** | **Of Special Interest** |
| --- | --- | --- | --- |
| **TP53 (V$P53F)** | 148 |  |  |
| **NFKB1 (V$NFKB)** | 144 |  | x |
| **JUN (V$AP1F)** | 123 |  |  |
| **ESR1 (V$EREF)** | 97 |  |  |
| **MYC (V$EBOX)** | 94 |  |  |
| **SP1 (V$SP1F)** | 88 |  |  |
| **STAT3 (V$STAT)** | 86 |  | x |
| **FOS (V$AP1F)** | 78 |  |  |
| **STAT1 (V$IRFF)** | 74 |  |  |
| **HIF1A (V$HIFF)** | 72 |  |  |
| **PPARG (V$PERO)** | 69 |  |  |
| **EGR1 (V$EGRF)** | 65 |  | x |
| **CREB1 (V$CREB)** | 63 |  |  |
| **E2F1 (V$E2FF)** | 60 |  |  |
| **PGR (V$GREF)** | 58 |  |  |
| **AR (V$GREF)** | 55 |  |  |
| **IRF1 (V$IRFF)** | 51 |  |  |
| **NR3C1 (V$GREF)** | 49 |  | x |
| **CEBPB (V$CEBP)** | 47 |  |  |
| **SMAD2 (V$SMAD)** | 46 |  |  |
| **VDR (V$RXRF)** | 46 |  |  |
| **FOXP3 (V$FKHD)** | 45 |  |  |
| **PPARA (V$PERO)** | 45 |  |  |
| **MYB (V$MYBL)** | 43 |  |  |
| **POU5F1 (V$STEM)** | 43 |  |  |
| **FOXO1 (V$FKHD)** | 41 |  |  |
| **LEF1 (V$LEFF)** | 41 |  |  |
| **SMAD4 (V$SMAD)** | 41 |  |  |
| **STAT5A (V$STAT)** | 41 |  |  |
| **JUNB (V$AP1F)** | 40 |  |  |
| **WT1 (V$EGRF)** | 40 |  |  |
| **ETS1 (V$ETSF)** | 39 |  |  |
| **ESR2 (V$EREF)** | 37 |  |  |
| **RUNX1 (V$HAML)** | 37 |  |  |
| **RUNX2 (V$HAML)** | 37 |  |  |
| **CEBPA (V$CEBP)** | 36 |  |  |
| **RARA (V$RXRF)** | 36 |  |  |
| **RELA (V$NFKB)** | 36 |  |  |
| **FOXO3 (V$FKHD)** | 35 |  |  |
| **GATA3 (V$GATA)** | 35 |  |  |
| **SPI1 (V$ETSF)** | 35 |  |  |
| **STAT6 (V$STAT)** | 35 |  |  |
| **PPARD (V$PERO)** | 34 |  |  |
| **SMAD3 (V$SMAD)** | 34 |  |  |
| **ATF3 (V$CREB)** | 33 |  |  |
| **MYCN (V$EBOX)** | 33 |  |  |
| **SOX2 (V$SORY)** | 33 |  |  |
| **TP73 (V$P53F)** | 33 |  |  |
| **ABL1 (V$CABL)** | 31 |  |  |
| **FOXM1 (V$FKHD)** | 31 | x | x |
| **KLF4 (V$KLFS)** | 31 |  |  |
| **FOSL1 (V$AP1F)** | 30 | x |  |
| **HNF4A (V$NR2F)** | 30 |  |  |
| **IRF3 (V$IRFF)** | 30 |  |  |
| **IRF4 (V$IRFF)** | 30 |  |  |
| **SOX9 (V$SORY)** | 30 |  |  |
