# Supplementary material for: The Molecular Phenotype of Endocapillary Proliferation: Novel Therapeutic Targets for IgA Nephropathy
Source: PLoS One. 2014 Aug 18;9(8):e103413. doi: 10.1371/journal.pone.0103413 (PMC4136785; doi:10.1371/journal.pone.0103413)
Supplement: Table S7 — Compounds from Connectivity map analysis results (p-value<0.05). These compounds are predicted to have favourable effects reversing the majority of differential mRNA expression changes associated with endocapillary proliferation. Compounds of interest are highlighted in grey. (DOCX) [file pone.0103413.s008.docx]

**Supplementary Table S7**. Compounds from Connectivity map analysis results (p-value<0.05). These compounds are predicted to have favourable effects reversing the majority of differential mRNA expression changes associated with endocapillary proliferation. Compounds of interest are highlighted in grey.

| **rank** | **cmap name** | **p** | **specificity** |
| --- | --- | --- | --- |
| 1 | **hydroquinine** | 0.0002 | 0.0000 |
| 2 | **atractyloside** | 0.0003 | 0.0056 |
| 3 | **resveratrol** | 0.0003 | 0.0463 |
| 4 | **alpha-yohimbine** | 0.0004 | 0.0000 |
| 5 | **propantheline bromide** | 0.0007 | 0.0000 |
| 6 | **theobromine** | 0.0007 | 0.0000 |
| 7 | **NS-398** | 0.0015 | 0.0063 |
| 8 | **guaifenesin** | 0.0016 | 0.0052 |
| 9 | **sulfachlorpyridazine** | 0.0021 | 0.0000 |
| 10 | **methotrexate** | 0.0025 | 0.0206 |
| 11 | **trihexyphenidyl** | 0.0025 | 0.0223 |
| 12 | **genistein** | 0.0061 | 0.1454 |
| 13 | **ciprofloxacin** | 0.0062 | 0.0000 |
| 14 | **nafcillin** | 0.0078 | 0.0000 |
| 15 | **hexetidine** | 0.0081 | 0.0407 |
| 16 | **isoniazid** | 0.0094 | 0.0199 |
| 17 | **clindamycin** | 0.0102 | 0.0073 |
| 18 | **nifurtimox** | 0.0103 | 0.0093 |
| 19 | **luteolin** | 0.0112 | 0.0457 |
| 20 | **clonidine** | 0.0131 | 0.0113 |
| 21 | **cinchonine** | 0.0133 | 0.1200 |
| 22 | **etoposide** | 0.0140 | 0.0855 |
| 23 | **ethisterone** | 0.0144 | 0.0000 |
| 24 | **ciclosporin** | 0.0148 | 0.0467 |
| 25 | **melatonin** | 0.0154 | 0.0256 |
| 26 | **chenodeoxycholic acid** | 0.0169 | 0.1154 |
| 27 | **monobenzone** | 0.0172 | 0.0338 |
| 28 | **CP-863187** | 0.0178 | 0.0471 |
| 29 | **carisoprodol** | 0.0186 | 0.0066 |
| 30 | **vinburnine** | 0.0188 | 0.0526 |
| 31 | **pralidoxime** | 0.0190 | 0.0355 |
| 32 | **Prestwick-1080** | 0.0193 | 0.0150 |
| 33 | **lomustine** | 0.0200 | 0.1479 |
| 34 | **parthenolide** | 0.0202 | 0.1517 |
| 35 | **geldanamycin** | 0.0204 | 0.4511 |
| 36 | **lycorine** | 0.0212 | 0.2407 |
| 37 | **chlorpromazine** | 0.0213 | 0.1026 |
| 38 | **chloramphenicol** | 0.0217 | 0.0208 |
| 39 | **piperlongumine** | 0.0221 | 0.0253 |
| 40 | **phenoxybenzamine** | 0.0221 | 0.2000 |
| 41 | **alvespimycin** | 0.0225 | 0.2644 |
| 42 | **econazole** | 0.0234 | 0.1111 |
| 43 | **6-bromoindirubin-3'-oxime** | 0.0235 | 0.1963 |
| 44 | **amitriptyline** | 0.0240 | 0.2282 |
| 45 | **ethosuximide** | 0.0245 | 0.0345 |
| 46 | **rimexolone** | 0.0251 | 0.0881 |
| 47 | **iproniazid** | 0.0264 | 0.0782 |
| 48 | **N-acetyl-L-aspartic acid** | 0.0268 | 0.0250 |
| 49 | **flupentixol** | 0.0270 | 0.0480 |
| 50 | **bupivacaine** | 0.0272 | 0.0185 |
| 51 | **MS-275** | 0.0273 | 0.1472 |
| 52 | **8-azaguanine** | 0.0282 | 0.1678 |
| 53 | **halofantrine** | 0.0290 | 0.0402 |
| 54 | **rotenone** | 0.0291 | 0.0526 |
| 55 | **paclitaxel** | 0.0293 | 0.0242 |
| 56 | **cefuroxime** | 0.0297 | 0.0526 |
| 57 | **vigabatrin** | 0.0298 | 0.0882 |
| 58 | **dextromethorphan** | 0.0306 | 0.0863 |
| 59 | **albendazole** | 0.0307 | 0.0349 |
| 60 | **amiodarone** | 0.0312 | 0.0898 |
| 61 | **nadolol** | 0.0321 | 0.3242 |
| 62 | **tubocurarine chloride** | 0.0321 | 0.0305 |
| 63 | **Prestwick-559** | 0.0323 | 0.0667 |
| 64 | **corticosterone** | 0.0324 | 0.0567 |
| 65 | **tiapride** | 0.0328 | 0.0294 |
| 66 | **iopamidol** | 0.0336 | 0.1384 |
| 67 | **piretanide** | 0.0369 | 0.0379 |
| 68 | **suxibuzone** | 0.0380 | 0.0365 |
| 69 | **cicloheximide** | 0.0380 | 0.2655 |
| 70 | **metrizamide** | 0.0395 | 0.0959 |
| 71 | **pivmecillinam** | 0.0397 | 0.0663 |
| 72 | **methylprednisolone** | 0.0405 | 0.0950 |
| 73 | **apomorphine** | 0.0406 | 0.1050 |
| 74 | **W-13** | 0.0406 | 0.0403 |
| 75 | **3-acetamidocoumarin** | 0.0418 | 0.2532 |
| 76 | **ciclopirox** | 0.0445 | 0.1714 |
| 77 | **carbimazole** | 0.0446 | 0.1325 |
| 78 | **zardaverine** | 0.0446 | 0.0725 |
| 79 | **pirenzepine** | 0.0461 | 0.0245 |
| 80 | **pinacidil** | 0.0464 | 0.0497 |
| 81 | **CP-944629** | 0.0482 | 0.0989 |
| 82 | **ronidazole** | 0.0484 | 0.1185 |
| 83 | **cyproterone** | 0.0500 | 0.1444 |
