# Supplementary material for: The Molecular Phenotype of Endocapillary Proliferation: Novel Therapeutic Targets for IgA Nephropathy
Source: PLoS One. 2014 Aug 18;9(8):e103413. doi: 10.1371/journal.pone.0103413 (PMC4136785; doi:10.1371/journal.pone.0103413)
Supplement: Table S8 — Drug pair seeker analysis. Top computationally-predicted drugs that would enhance the reversal of gene expression changes associated with endocapillary proliferation when combined with methylprednisolone or corticosterone. Coverage = number of desirable targets that the drug affects, meaning the transcripts described in endocapillary proliferation that the drug would reverse. Conflict = the number of genes the drug is potentially changing in an undesired direction in endocapillary proliferation. (DOCX) [file pone.0103413.s009.docx]

**Supplementary Table S8.**  Drug pair seeker analysis. Top computationally-predicted drugs that would enhance the reversal of gene expression changes associated with endocapillary proliferation when combined with methylprednisolone or corticosterone. Coverage = number of desirable targets that the drug affects, meaning the transcripts described in endocapillary proliferation that the drug would reverse. Conflict = the number of genes the drug is potentially changing in an undesired direction in endocapillary proliferation.

| **Drug 1** | **Drug 2** | Total Coverage | Total Conflicts | Drug 1 Coverage | Drug 1 Conflicts | Drug 2 Coverage | Drug 2 Conflicts |
| --- | --- | --- | --- | --- | --- | --- | --- |
| methylprednisolone-7137 | resveratrol-622 | 51 | 11 | 9 | 6 | 42 | 5 |
| methylprednisolone-7137 | monobenzone-5312 | 49 | 9 | 9 | 6 | 40 | 4 |
| methylprednisolone-6785 | resveratrol-622 | 47 | 7 | 5 | 2 | 42 | 5 |
| methylprednisolone-3183 | resveratrol-622 | 51 | 13 | 10 | 8 | 42 | 5 |
| methylprednisolone-3183 | monobenzone-5312 | 49 | 11 | 10 | 8 | 40 | 4 |
| methylprednisolone-7137 | etoposide-3241 | 51 | 13 | 9 | 6 | 42 | 8 |
| methylprednisolone-6785 | etoposide-3241 | 47 | 9 | 5 | 2 | 42 | 8 |
| methylprednisolone-6785 | etoposide-5027 | 47 | 9 | 5 | 2 | 42 | 7 |
| methylprednisolone-6785 | monobenzone-5312 | 44 | 6 | 5 | 2 | 40 | 4 |
| corticosterone-4145 | resveratrol-622 | 47 | 11 | 6 | 6 | 42 | 5 |
| corticosterone-4145 | etoposide-5027 | 47 | 12 | 6 | 6 | 42 | 7 |
| corticosterone-4145 | monobenzone-5312 | 45 | 10 | 6 | 6 | 40 | 4 |
| corticosterone-4145 | etoposide-3241 | 48 | 14 | 6 | 6 | 42 | 8 |
| corticosterone-4145 | resveratrol-958 | 43 | 9 | 6 | 6 | 38 | 3 |
| corticosterone-3244 | resveratrol-622 | 46 | 12 | 4 | 7 | 42 | 5 |
| corticosterone-3244 | etoposide-5027 | 46 | 13 | 4 | 7 | 42 | 7 |
| corticosterone-3244 | monobenzone-5312 | 44 | 11 | 4 | 7 | 40 | 4 |
| corticosterone-4064 | resveratrol-622 | 43 | 10 | 1 | 5 | 42 | 5 |
